# Supplementary material for: Textbook-level medical knowledge in large language models: comparative evaluation using Japanese National Medical Examination
Source: BMC Med Inform Decis Mak. 2026 Feb 3;26:65. doi: 10.1186/s12911-026-03370-y (PMC12958580; doi:10.1186/s12911-026-03370-y)
Supplement: Supplementary file 2 — Supplementary Material 2 [file 12911_2026_3370_MOESM2_ESM.pptx]

## Slide 1
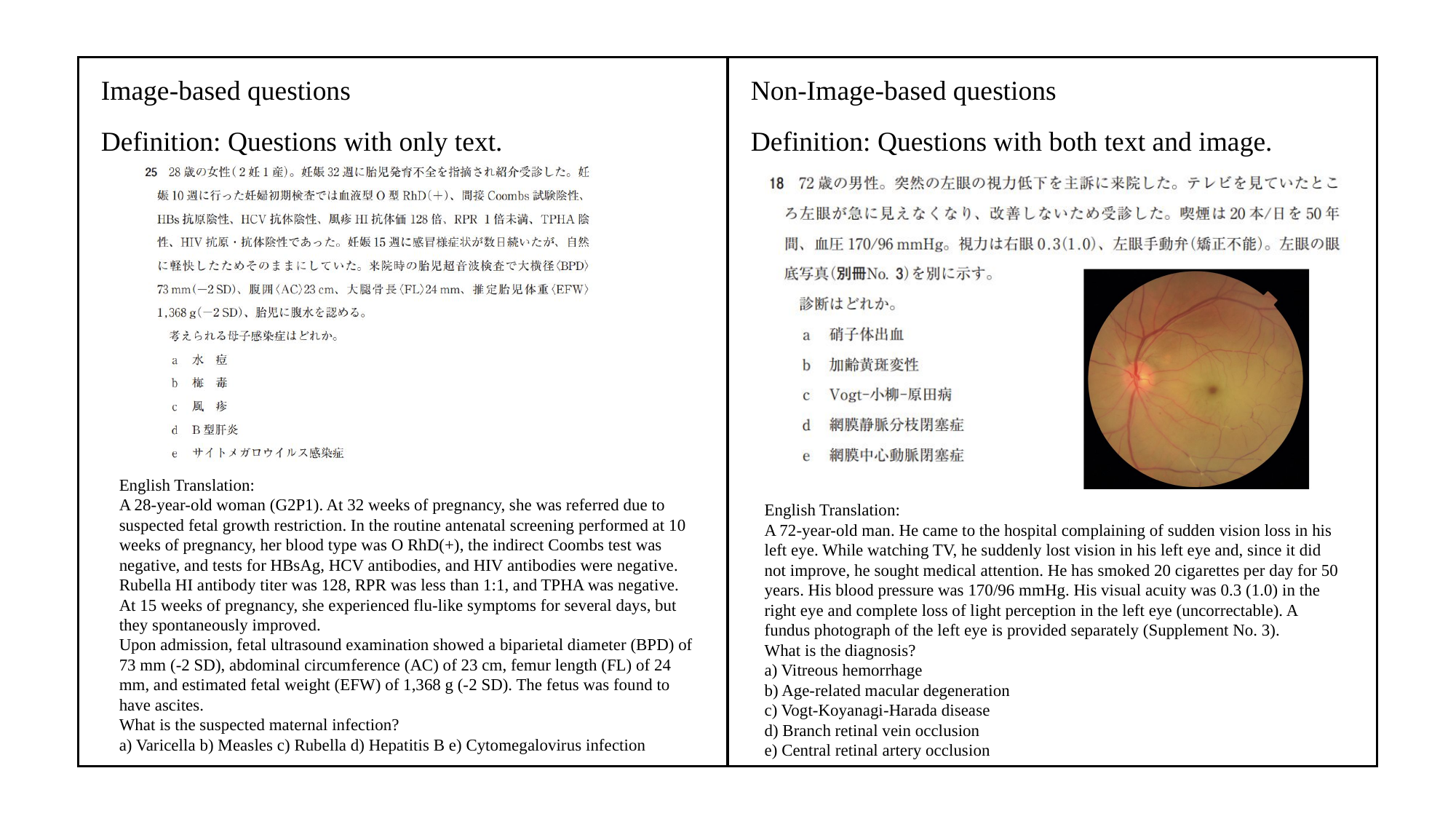

Image-based questions
Non-Image-based questions
Definition: Questions with only text.
Definition: Questions with both text and image.
English Translation:
A 28-year-old woman (G2P1). At 32 weeks of pregnancy, she was referred due to suspected fetal growth restriction. In the routine antenatal screening performed at 10 weeks of pregnancy, her blood type was O RhD(+), the indirect Coombs test was negative, and tests for HBsAg, HCV antibodies, and HIV antibodies were negative. Rubella HI antibody titer was 128, RPR was less than 1:1, and TPHA was negative. At 15 weeks of pregnancy, she experienced flu-like symptoms for several days, but they spontaneously improved.
Upon admission, fetal ultrasound examination showed a biparietal diameter (BPD) of 73 mm (-2 SD), abdominal circumference (AC) of 23 cm, femur length (FL) of 24 mm, and estimated fetal weight (EFW) of 1,368 g (-2 SD). The fetus was found to have ascites.
What is the suspected maternal infection?
a) Varicella b) Measles c) Rubella d) Hepatitis B e) Cytomegalovirus infection
English Translation:
A 72-year-old man. He came to the hospital complaining of sudden vision loss in his left eye. While watching TV, he suddenly lost vision in his left eye and, since it did not improve, he sought medical attention. He has smoked 20 cigarettes per day for 50 years. His blood pressure was 170/96 mmHg. His visual acuity was 0.3 (1.0) in the right eye and complete loss of light perception in the left eye (uncorrectable). A fundus photograph of the left eye is provided separately (Supplement No. 3).
What is the diagnosis?
a) Vitreous hemorrhage
b) Age-related macular degeneration
c) Vogt-Koyanagi-Harada disease
d) Branch retinal vein occlusion
e) Central retinal artery occlusion

## Slide 2
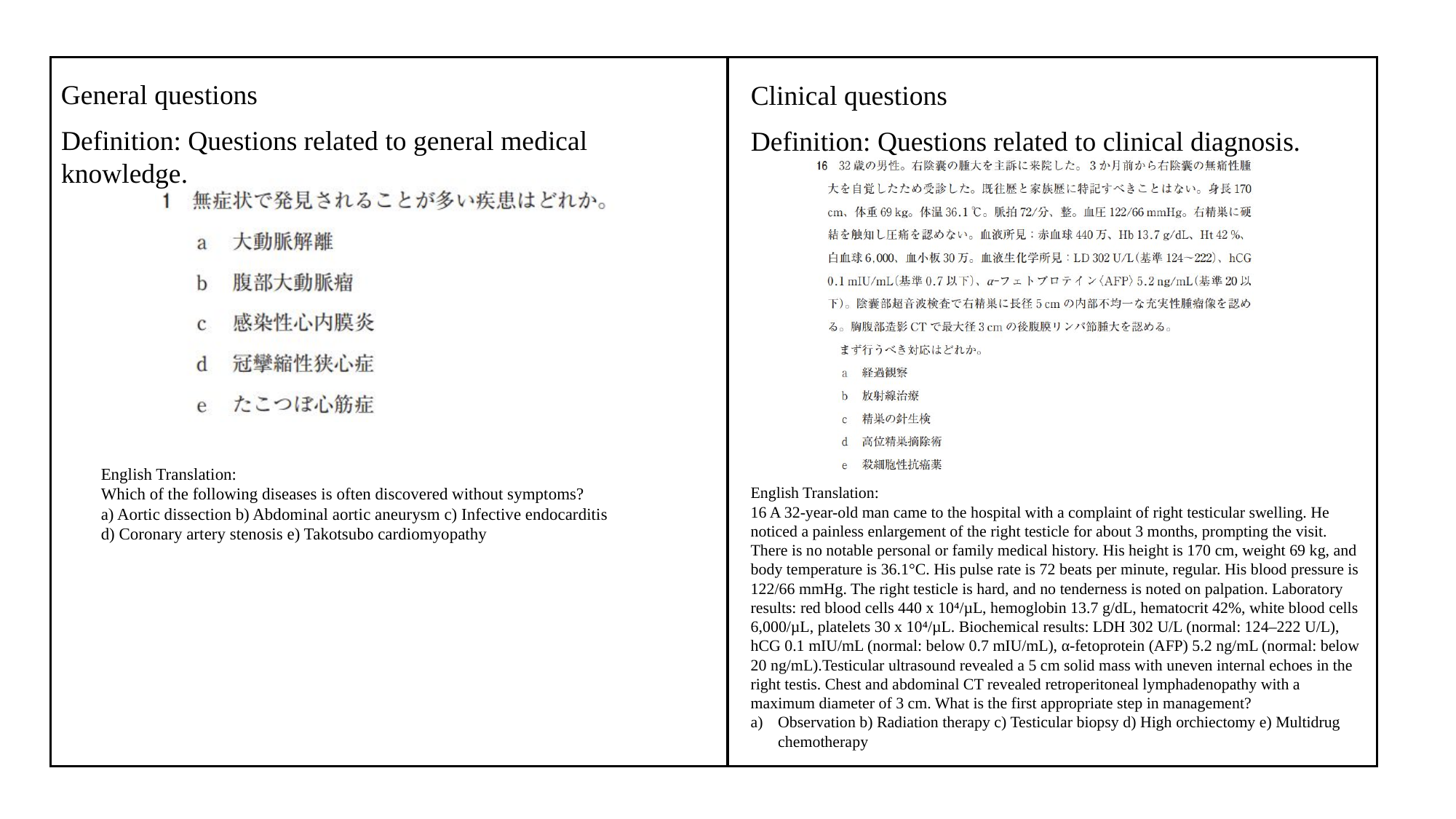

General questions
Clinical questions
Definition: Questions related to general medical knowledge.
Definition: Questions related to clinical diagnosis.
English Translation:
Which of the following diseases is often discovered without symptoms?
a) Aortic dissection b) Abdominal aortic aneurysm c) Infective endocarditis
d) Coronary artery stenosis e) Takotsubo cardiomyopathy
English Translation:
16 A 32-year-old man came to the hospital with a complaint of right testicular swelling. He noticed a painless enlargement of the right testicle for about 3 months, prompting the visit. There is no notable personal or family medical history. His height is 170 cm, weight 69 kg, and body temperature is 36.1°C. His pulse rate is 72 beats per minute, regular. His blood pressure is 122/66 mmHg. The right testicle is hard, and no tenderness is noted on palpation. Laboratory results: red blood cells 440 x 10⁴/µL, hemoglobin 13.7 g/dL, hematocrit 42%, white blood cells 6,000/µL, platelets 30 x 10⁴/µL. Biochemical results: LDH 302 U/L (normal: 124–222 U/L), hCG 0.1 mIU/mL (normal: below 0.7 mIU/mL), α-fetoprotein (AFP) 5.2 ng/mL (normal: below 20 ng/mL).Testicular ultrasound revealed a 5 cm solid mass with uneven internal echoes in the right testis. Chest and abdominal CT revealed retroperitoneal lymphadenopathy with a maximum diameter of 3 cm. What is the first appropriate step in management?
Observation b) Radiation therapy c) Testicular biopsy d) High orchiectomy e) Multidrug chemotherapy

## Slide 3
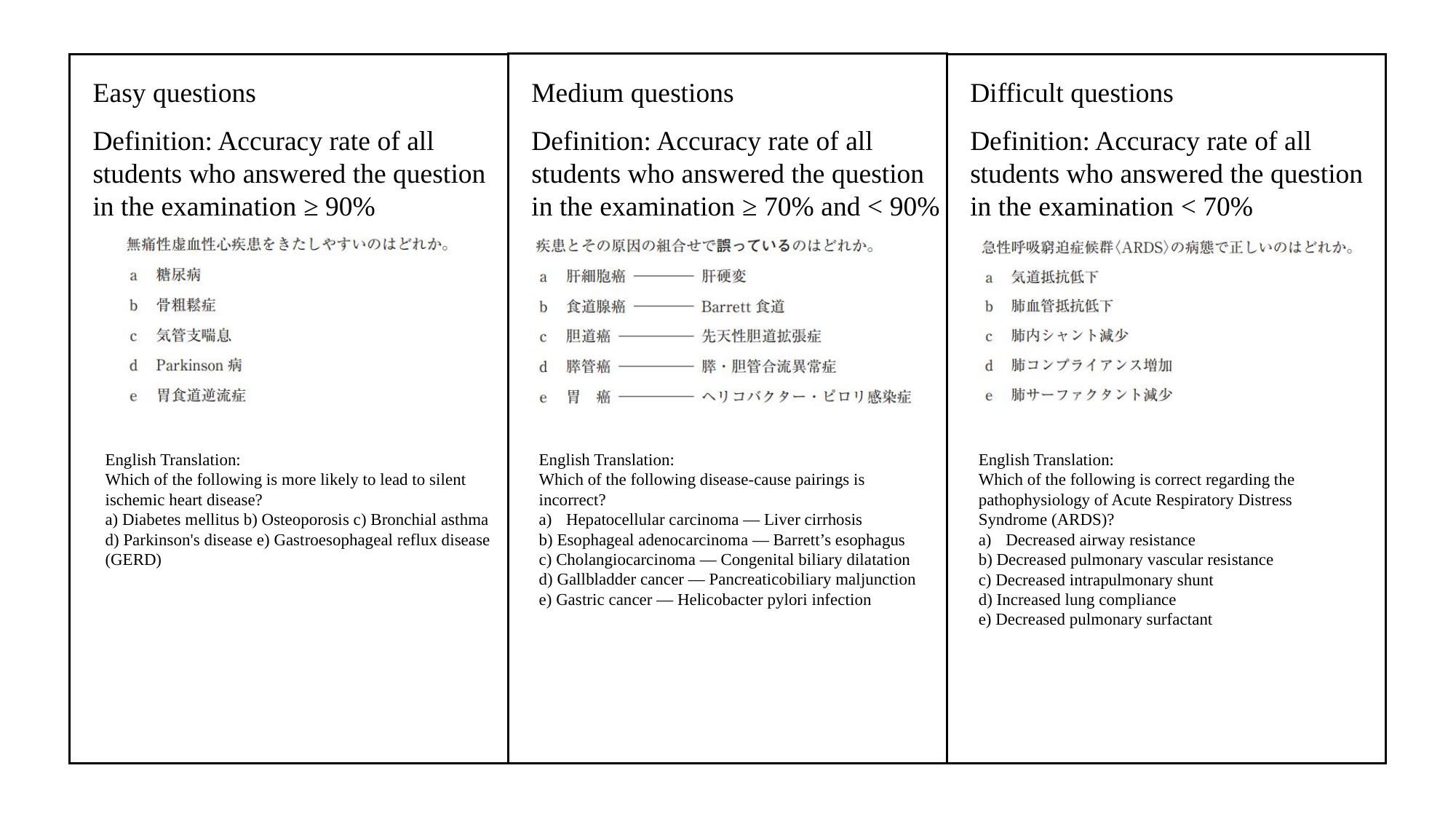

Difficult questions
Easy questions
Medium questions
Definition: Accuracy rate of all students who answered the question in the examination ≥ 70% and < 90%
Definition: Accuracy rate of all students who answered the question in the examination < 70%
Definition: Accuracy rate of all students who answered the question in the examination ≥ 90%
English Translation:
Which of the following is more likely to lead to silent ischemic heart disease?
a) Diabetes mellitus b) Osteoporosis c) Bronchial asthma d) Parkinson's disease e) Gastroesophageal reflux disease (GERD)
English Translation:
Which of the following disease-cause pairings is incorrect?
Hepatocellular carcinoma — Liver cirrhosis
b) Esophageal adenocarcinoma — Barrett’s esophagus
c) Cholangiocarcinoma — Congenital biliary dilatation
d) Gallbladder cancer — Pancreaticobiliary maljunction
e) Gastric cancer — Helicobacter pylori infection
English Translation:
Which of the following is correct regarding the pathophysiology of Acute Respiratory Distress Syndrome (ARDS)?
Decreased airway resistance
b) Decreased pulmonary vascular resistance
c) Decreased intrapulmonary shunt
d) Increased lung compliance
e) Decreased pulmonary surfactant
