# Supplementary material for: Textbook-level medical knowledge in large language models: comparative evaluation using Japanese National Medical Examination
Source: BMC Med Inform Decis Mak. 2026 Feb 3;26:65. doi: 10.1186/s12911-026-03370-y (PMC12958580; doi:10.1186/s12911-026-03370-y)
Supplement: Supplementary file 3 — Supplementary Material 3 [file 12911_2026_3370_MOESM3_ESM.pptx]

## Slide 1
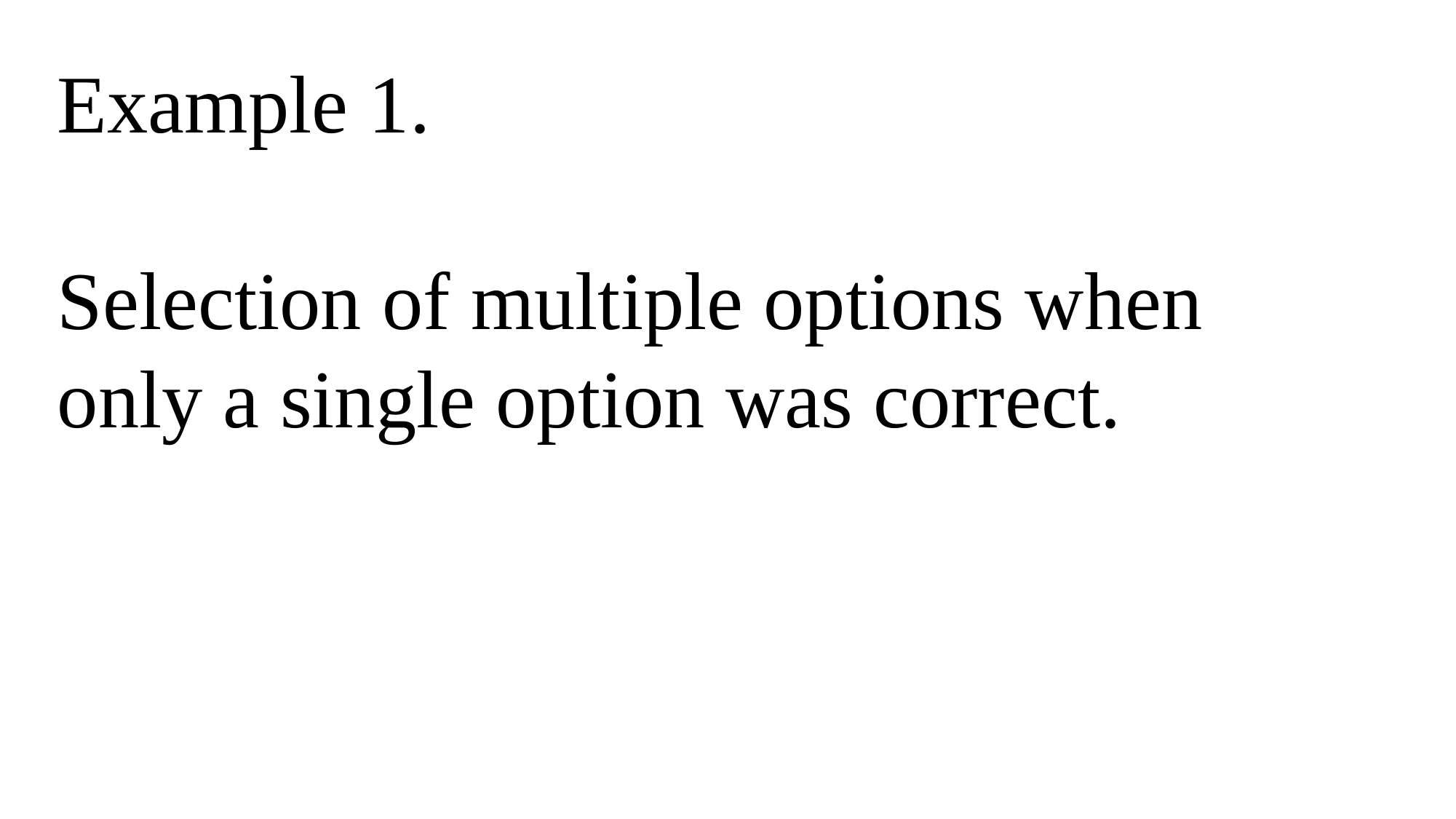

Example 1.
Selection of multiple options when only a single option was correct.

## Slide 2
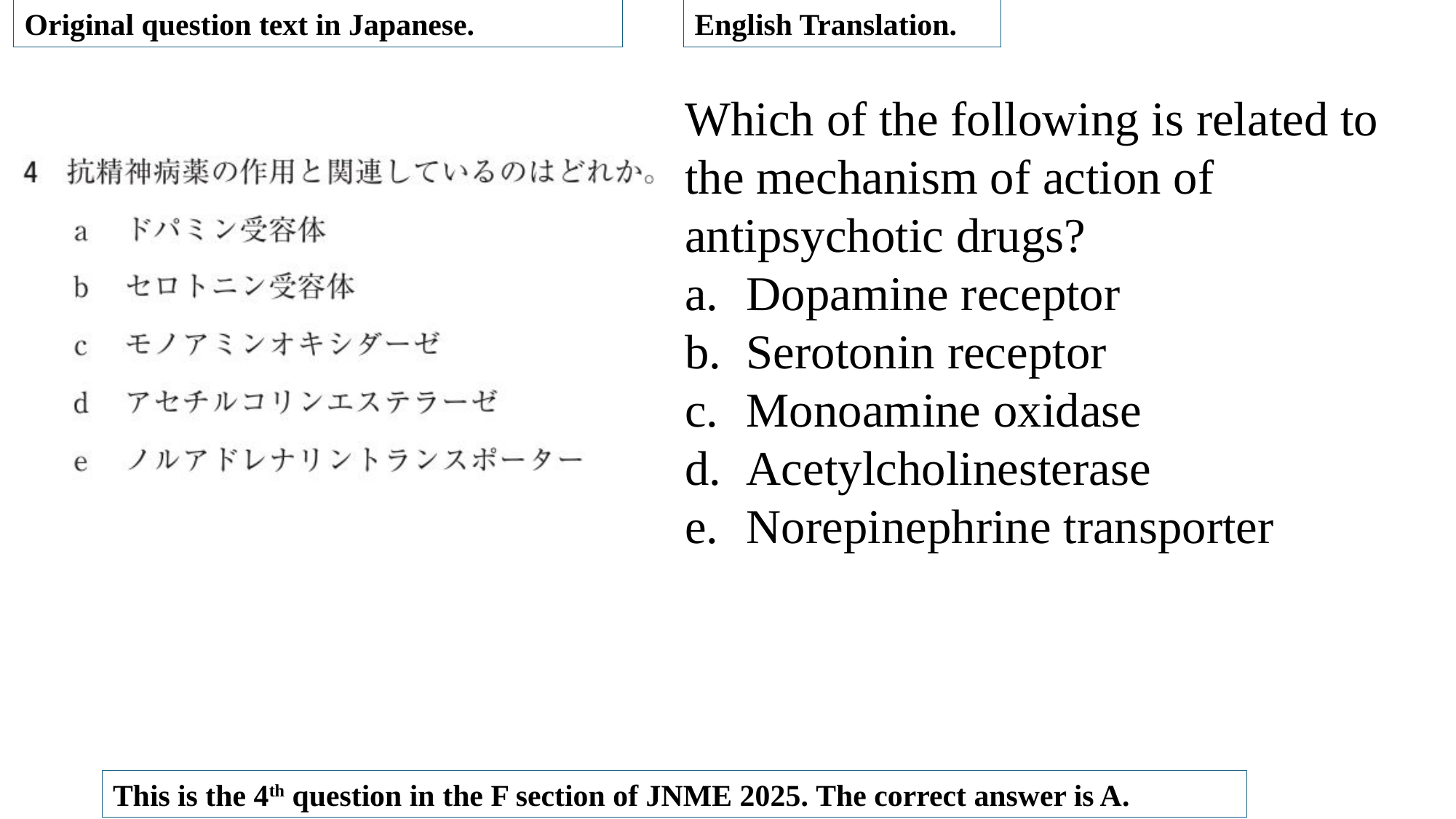

Original question text in Japanese.
English Translation.
Which of the following is related to the mechanism of action of antipsychotic drugs?
Dopamine receptor
Serotonin receptor
Monoamine oxidase
Acetylcholinesterase
Norepinephrine transporter
This is the 4th question in the F section of JNME 2025. The correct answer is A.

## Slide 3
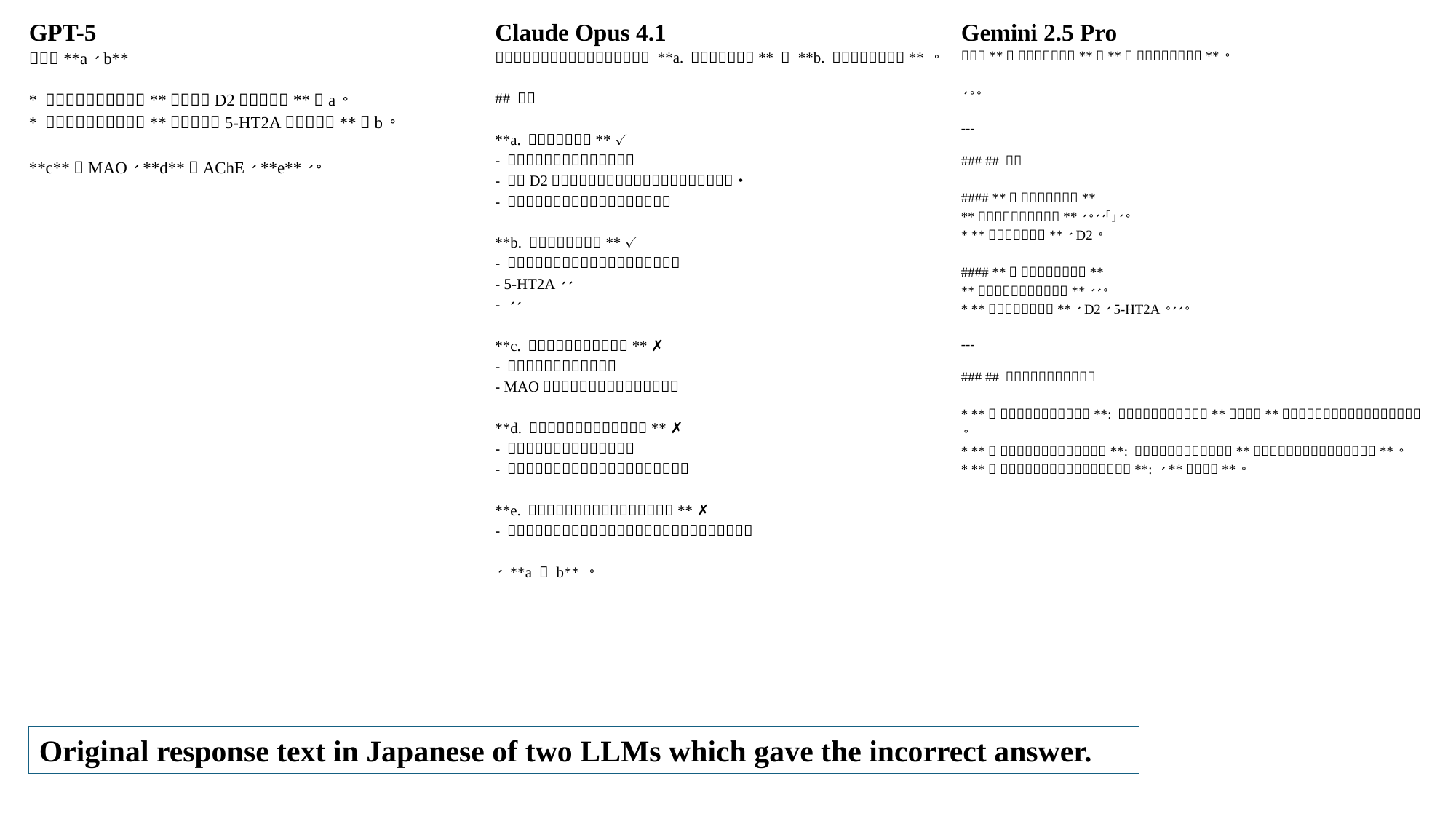

| GPT-5 正解：\*\*a、b\*\* \* 抗精神病薬の主作用は\*\*ドパミンD2受容体遮断\*\*（a）。 \* 非定型抗精神病薬では\*\*セロトニン5-HT2A受容体遮断\*\*（b）も重要。 \*\*c\*\*（MAO）、\*\*d\*\*（AChE）、\*\*e\*\*（ノルアドレナリントランスポーター）は主に抗うつ薬や認知症治療薬の標的で、抗精神病薬の主要機序ではありません。 | Claude Opus 4.1 抗精神病薬の作用と関連しているのは \*\*a. ドパミン受容体\*\* と \*\*b. セロトニン受容体\*\* です。 ## 解説 \*\*a. ドパミン受容体\*\* ✓ - 抗精神病薬の最も重要な作用点 - 特にD2受容体の遮断が統合失調症の陽性症状（幻覚・妄想）の改善に重要 - すべての抗精神病薬に共通する作用機序 \*\*b. セロトニン受容体\*\* ✓ - 特に第二世代（非定型）抗精神病薬で重要 - 5-HT2A受容体の遮断により、錐体外路症状が軽減され、陰性症状への効果も期待される - リスペリドン、オランザピン、クエチアピンなどが該当 \*\*c. モノアミンオキシダーゼ\*\* ✗ - モノアミンを分解する酵素 - MAO阻害薬は主に抗うつ薬として使用 \*\*d. アセチルコリンエステラーゼ\*\* ✗ - アセチルコリンを分解する酵素 - この酵素の阻害薬は認知症治療薬として使用 \*\*e. ノルアドレナリントランスポーター\*\* ✗ - ノルアドレナリン再取り込み阻害は主に抗うつ薬の作用機序 したがって、正解は \*\*a と b\*\* です。 | Gemini 2.5 Pro 正解は\*\*ａ ドパミン受容体\*\*と\*\*ｂ セロトニン受容体\*\*です。 抗精神病薬の主な作用機序は、脳内の神経伝達物質のバランスを調整することにあります。特にドパミンとセロトニンが重要な役割を果たしています。 --- ### ## 解説 #### \*\*ａ ドパミン受容体\*\* \*\*ドパミン受容体の遮断\*\*は、すべての抗精神病薬に共通する基本的な作用です。特に、統合失調症の陽性症状（幻覚や妄想など）は、脳内のドパミン神経系の過剰な活動が原因であるとする「ドパミン仮説」に基づいており、この受容体をブロックすることで症状を緩和します。 \* \*\*定型抗精神病薬\*\*（第一世代）は、主にドパミンD2受容体を強力に遮断します。 #### \*\*ｂ セロトニン受容体\*\* \*\*セロトニン受容体の遮断\*\*も、多くの抗精神病薬、特に新しいタイプの薬において重要な作用です。 \* \*\*非定型抗精神病薬\*\*（第二世代）は、ドパミンD2受容体に加え、セロトニン5-HT2A受容体などを遮断します。このセロトニン受容体への作用により、ドパミン遮断による副作用（錐体外路症状など）が軽減され、陰性症状（意欲の低下や感情の平板化など）への効果も期待されています。 --- ### ## その他の選択肢について \* \*\*ｃ モノアミンオキシダーゼ\*\*: この酵素を阻害する薬は\*\*抗うつ薬\*\*（モノアミンオキシダーゼ阻害薬）として用いられます。 \* \*\*ｄ アセチルコリンエステラーゼ\*\*: この酵素を阻害する薬は、\*\*アルツハイマー型認知症の治療薬\*\*として用いられます。 \* \*\*ｅ ノルアドレナリントランスポーター\*\*: これを阻害する薬は、主に\*\*抗うつ薬\*\*として用いられます。 |
| --- | --- | --- |
Original response text in Japanese of two LLMs which gave the incorrect answer.

## Slide 4
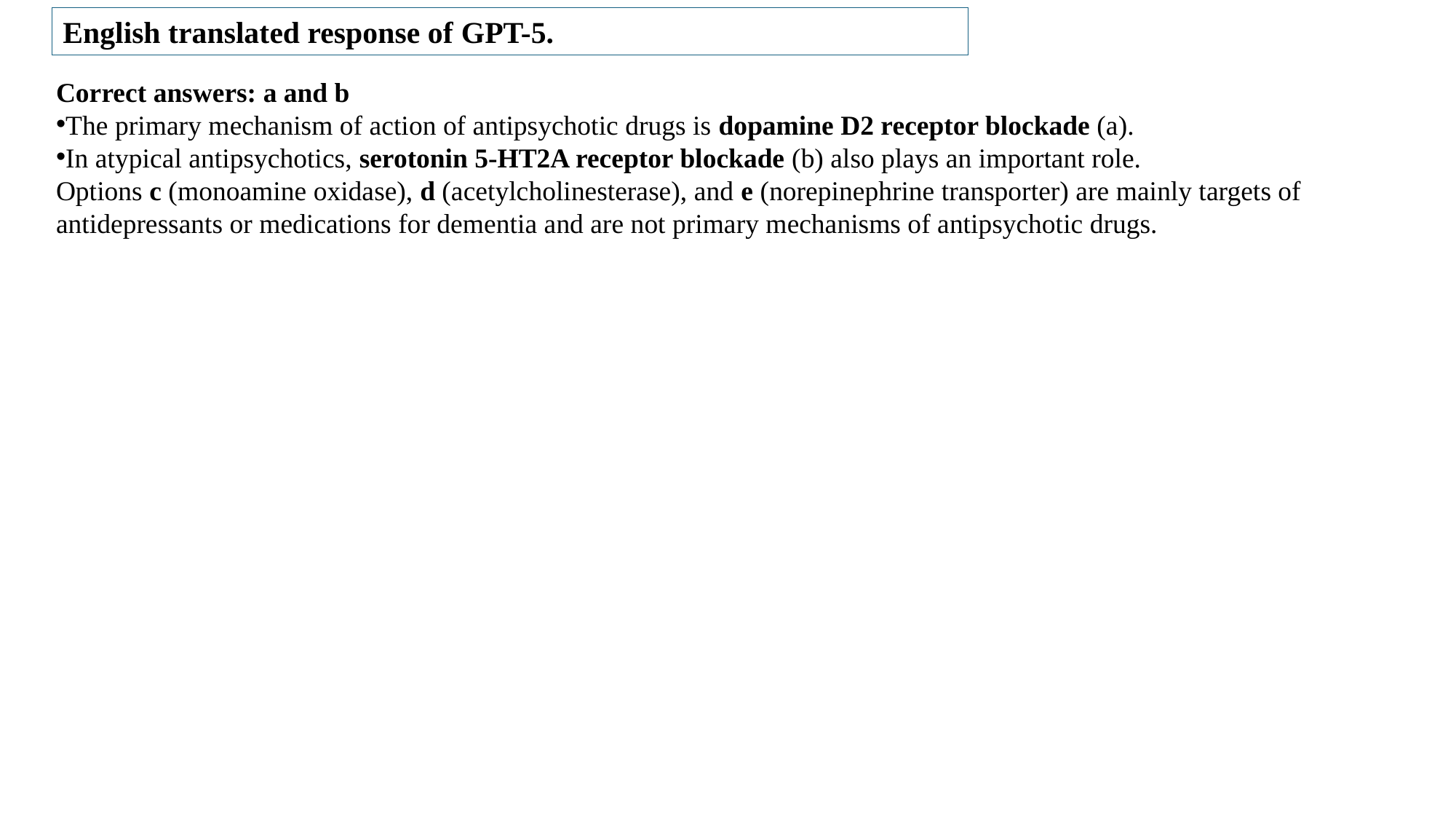

English translated response of GPT-5.
Correct answers: a and b
The primary mechanism of action of antipsychotic drugs is dopamine D2 receptor blockade (a).
In atypical antipsychotics, serotonin 5-HT2A receptor blockade (b) also plays an important role.
Options c (monoamine oxidase), d (acetylcholinesterase), and e (norepinephrine transporter) are mainly targets of antidepressants or medications for dementia and are not primary mechanisms of antipsychotic drugs.

## Slide 5
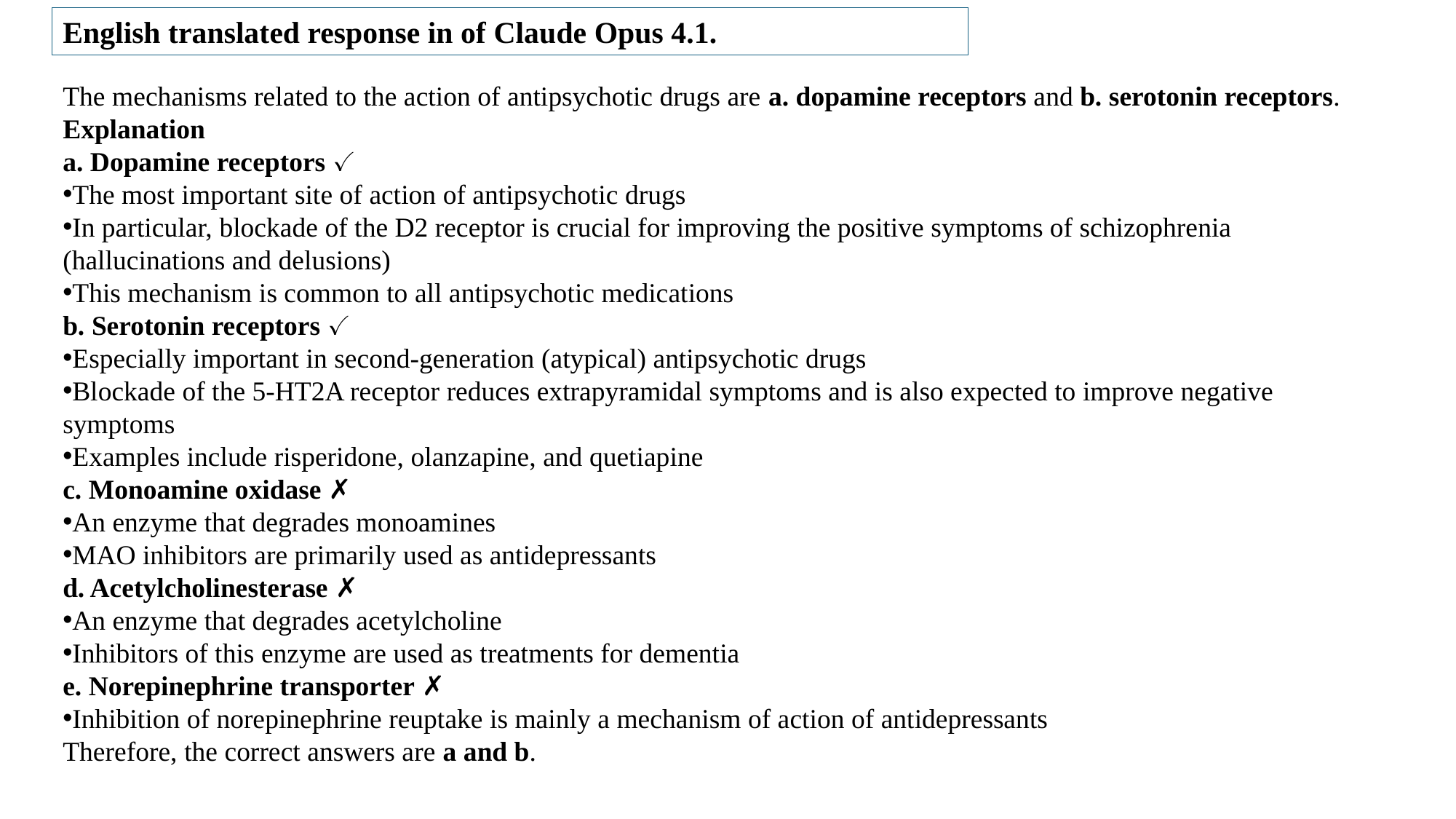

English translated response in of Claude Opus 4.1.
The mechanisms related to the action of antipsychotic drugs are a. dopamine receptors and b. serotonin receptors.
Explanation
a. Dopamine receptors ✓
The most important site of action of antipsychotic drugs
In particular, blockade of the D2 receptor is crucial for improving the positive symptoms of schizophrenia (hallucinations and delusions)
This mechanism is common to all antipsychotic medications
b. Serotonin receptors ✓
Especially important in second-generation (atypical) antipsychotic drugs
Blockade of the 5-HT2A receptor reduces extrapyramidal symptoms and is also expected to improve negative symptoms
Examples include risperidone, olanzapine, and quetiapine
c. Monoamine oxidase ✗
An enzyme that degrades monoamines
MAO inhibitors are primarily used as antidepressants
d. Acetylcholinesterase ✗
An enzyme that degrades acetylcholine
Inhibitors of this enzyme are used as treatments for dementia
e. Norepinephrine transporter ✗
Inhibition of norepinephrine reuptake is mainly a mechanism of action of antidepressants
Therefore, the correct answers are a and b.

## Slide 6
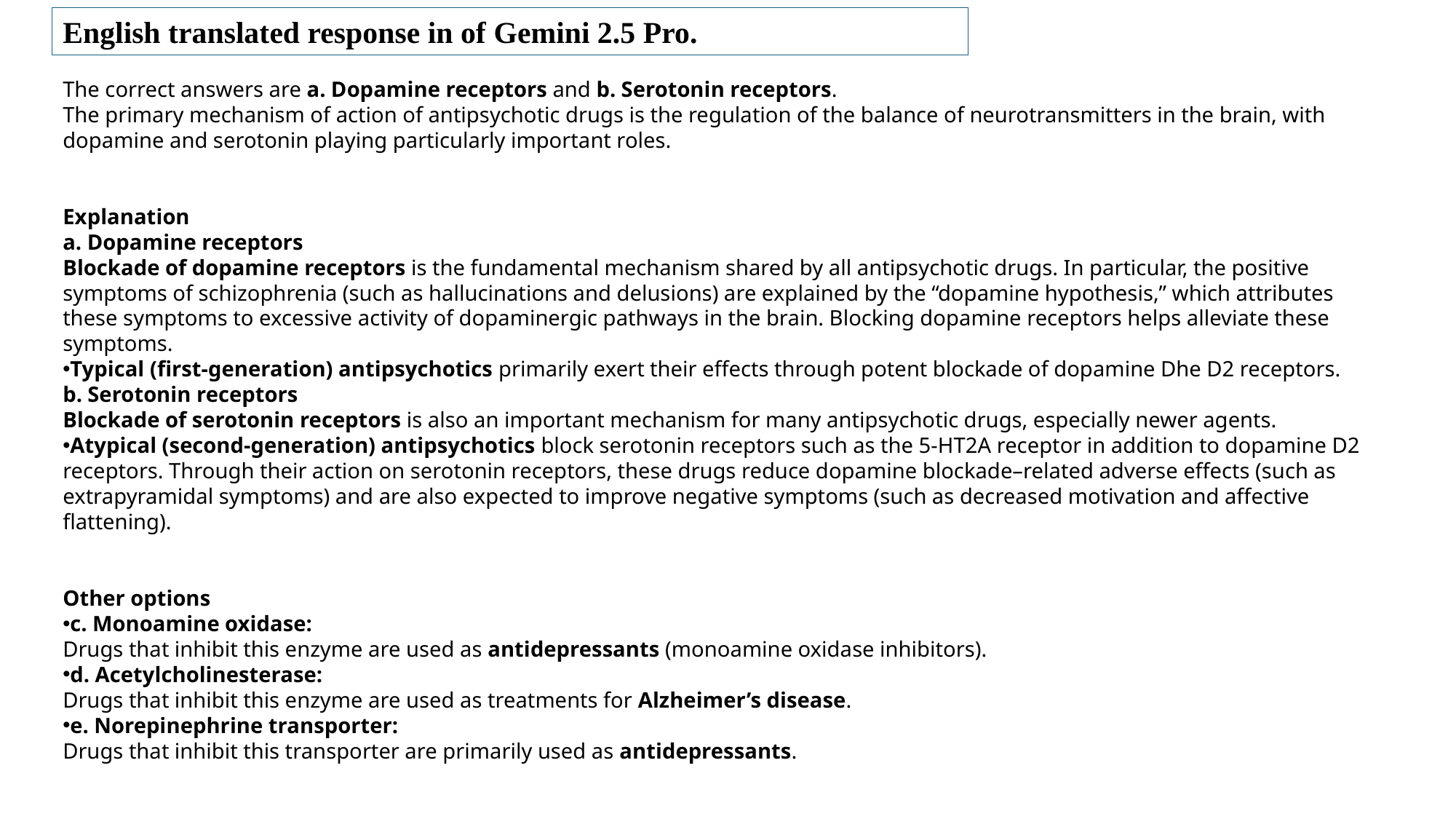

English translated response in of Gemini 2.5 Pro.
The correct answers are a. Dopamine receptors and b. Serotonin receptors.
The primary mechanism of action of antipsychotic drugs is the regulation of the balance of neurotransmitters in the brain, with dopamine and serotonin playing particularly important roles.
Explanation
a. Dopamine receptors
Blockade of dopamine receptors is the fundamental mechanism shared by all antipsychotic drugs. In particular, the positive symptoms of schizophrenia (such as hallucinations and delusions) are explained by the “dopamine hypothesis,” which attributes these symptoms to excessive activity of dopaminergic pathways in the brain. Blocking dopamine receptors helps alleviate these symptoms.
Typical (first-generation) antipsychotics primarily exert their effects through potent blockade of dopamine Dhe D2 receptors.
b. Serotonin receptors
Blockade of serotonin receptors is also an important mechanism for many antipsychotic drugs, especially newer agents.
Atypical (second-generation) antipsychotics block serotonin receptors such as the 5-HT2A receptor in addition to dopamine D2 receptors. Through their action on serotonin receptors, these drugs reduce dopamine blockade–related adverse effects (such as extrapyramidal symptoms) and are also expected to improve negative symptoms (such as decreased motivation and affective flattening).
Other options
c. Monoamine oxidase:
Drugs that inhibit this enzyme are used as antidepressants (monoamine oxidase inhibitors).
d. Acetylcholinesterase:
Drugs that inhibit this enzyme are used as treatments for Alzheimer’s disease.
e. Norepinephrine transporter:
Drugs that inhibit this transporter are primarily used as antidepressants.

## Slide 7
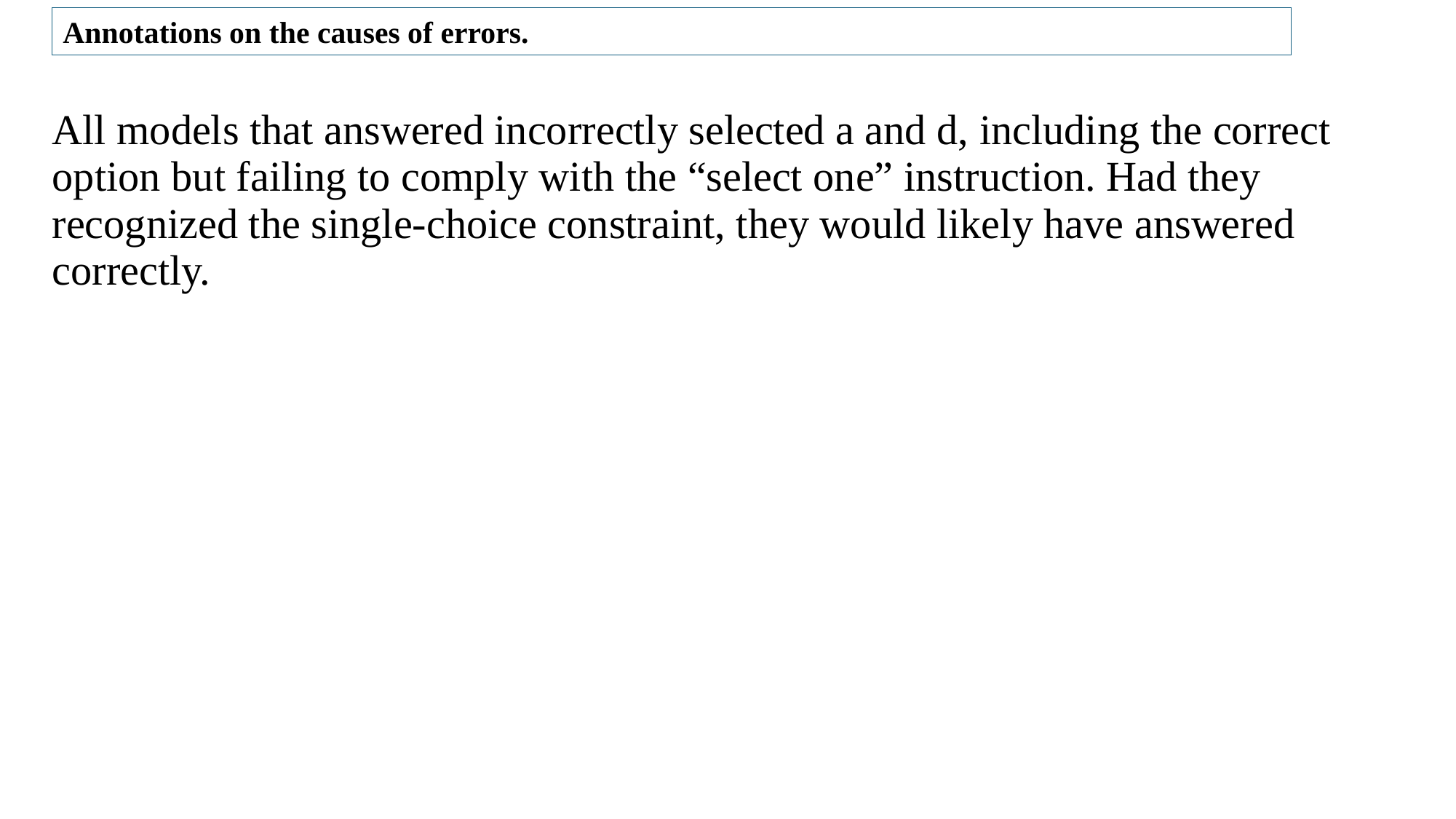

Annotations on the causes of errors.
| All models that answered incorrectly selected a and d, including the correct option but failing to comply with the “select one” instruction. Had they recognized the single-choice constraint, they would likely have answered correctly. |
| --- |

## Slide 8
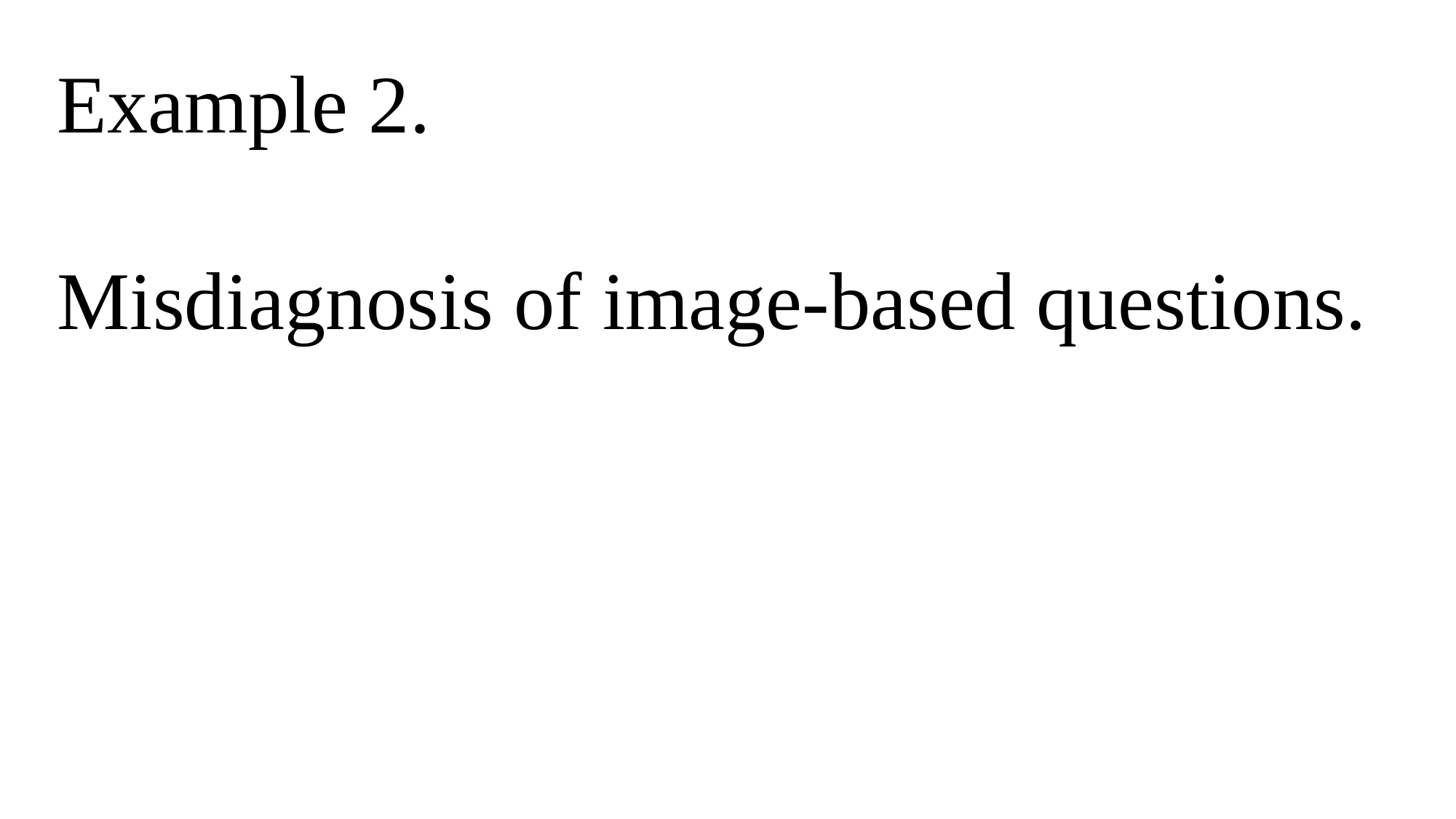

Example 2.
Misdiagnosis of image-based questions.

## Slide 9
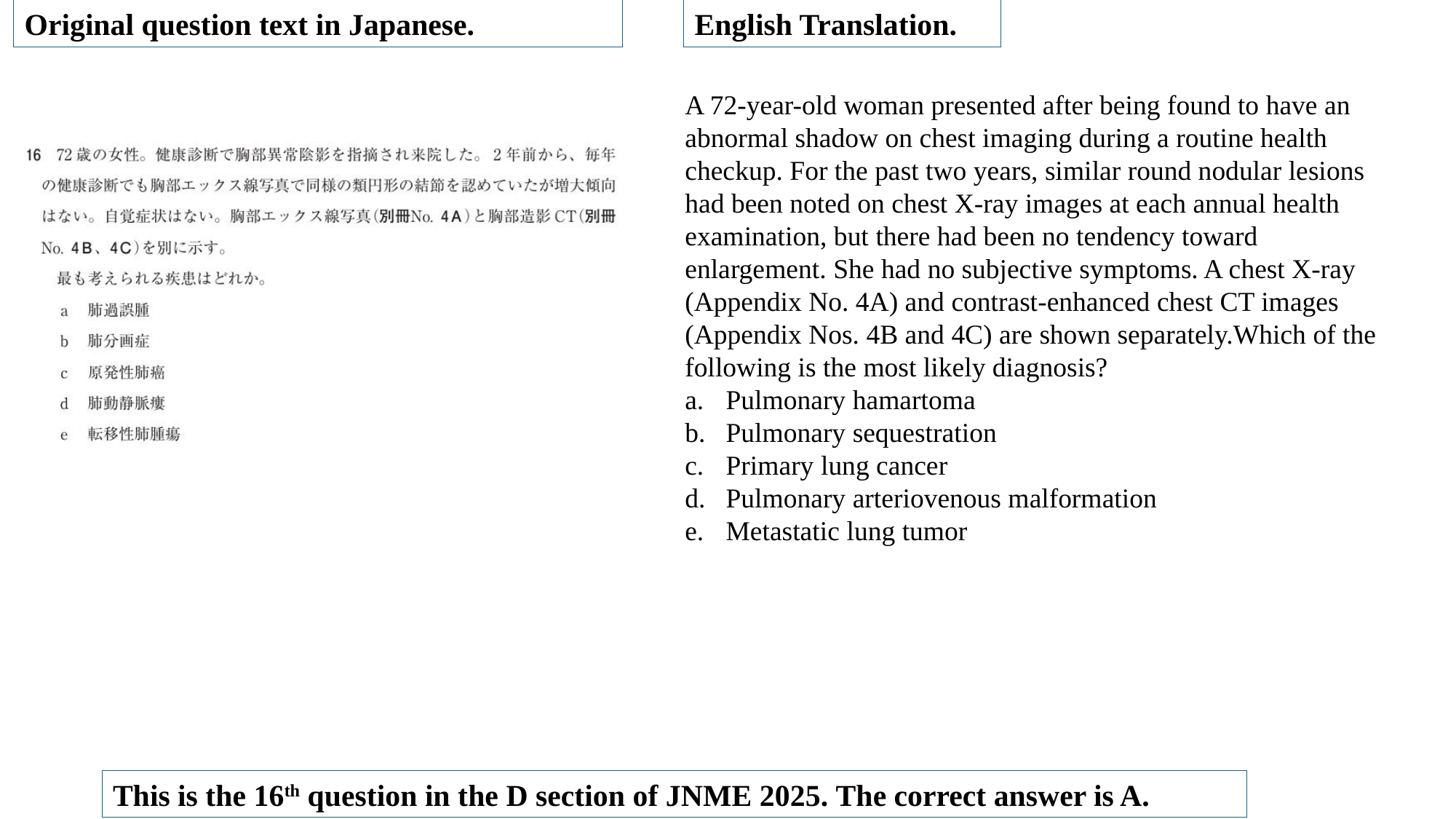

Original question text in Japanese.
English Translation.
A 72-year-old woman presented after being found to have an abnormal shadow on chest imaging during a routine health checkup. For the past two years, similar round nodular lesions had been noted on chest X-ray images at each annual health examination, but there had been no tendency toward enlargement. She had no subjective symptoms. A chest X-ray (Appendix No. 4A) and contrast-enhanced chest CT images (Appendix Nos. 4B and 4C) are shown separately.Which of the following is the most likely diagnosis?
Pulmonary hamartoma
Pulmonary sequestration
Primary lung cancer
Pulmonary arteriovenous malformation
Metastatic lung tumor
This is the 16th question in the D section of JNME 2025. The correct answer is A.

## Slide 10
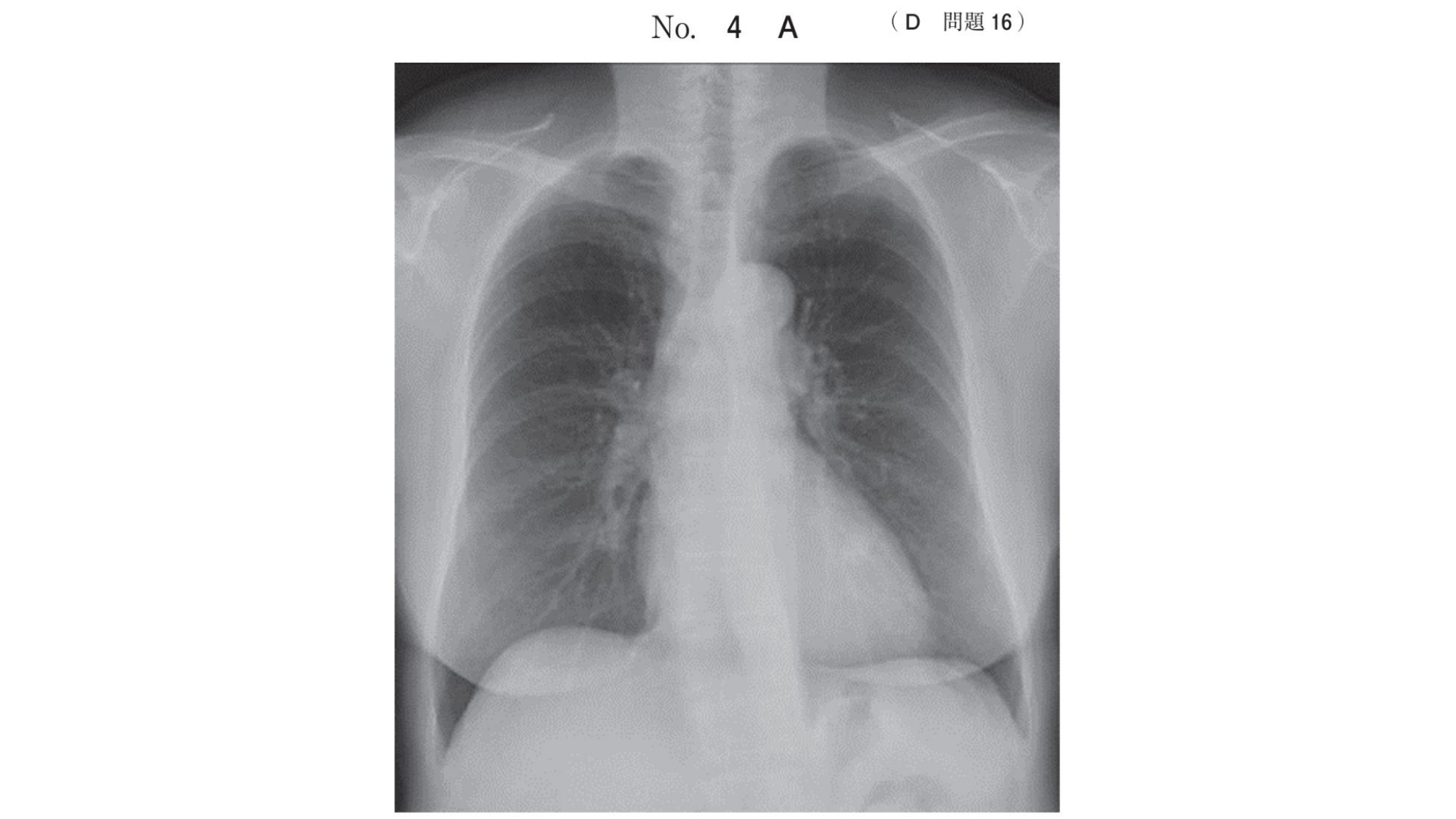

## Slide 11
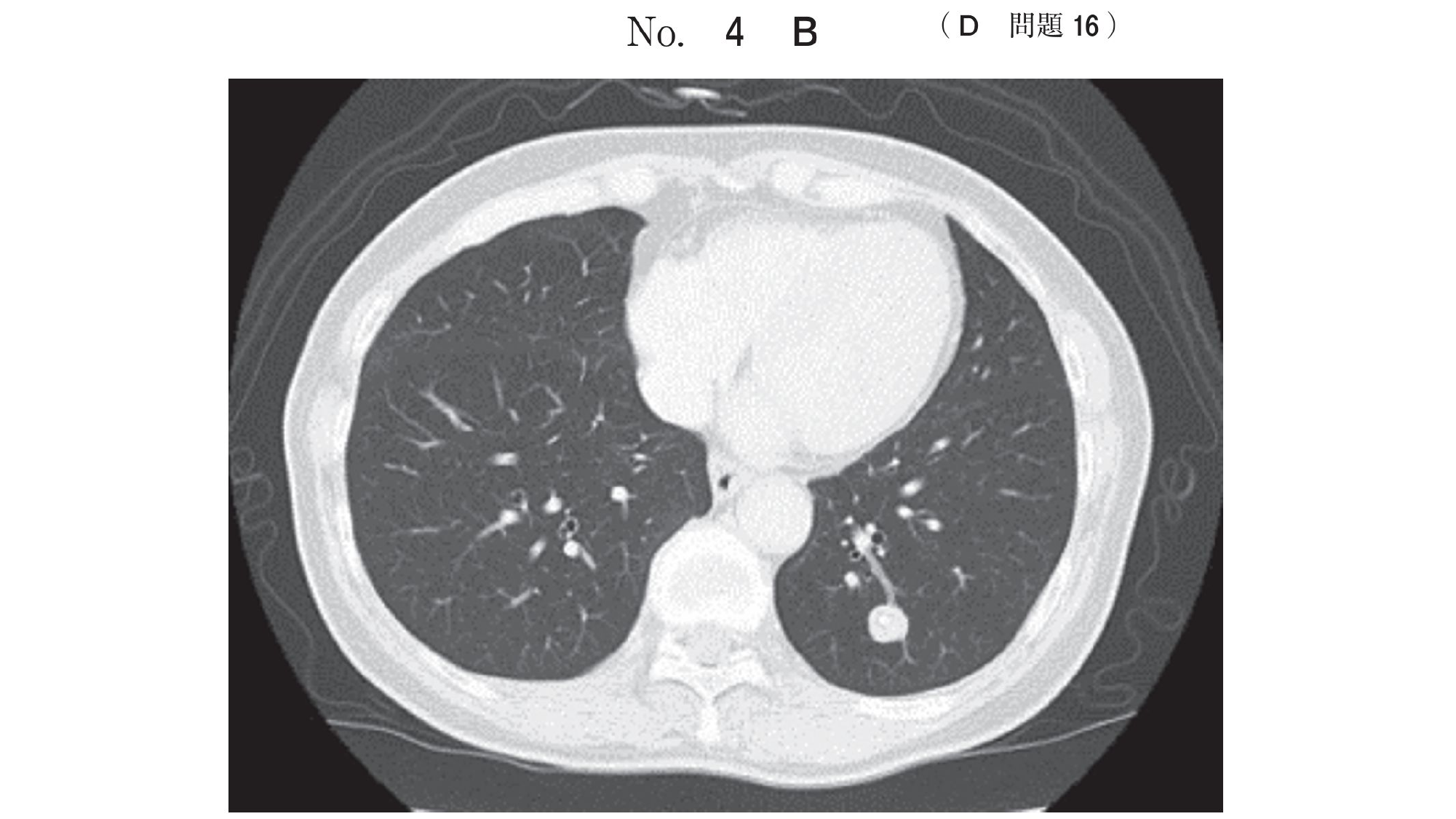

## Slide 12
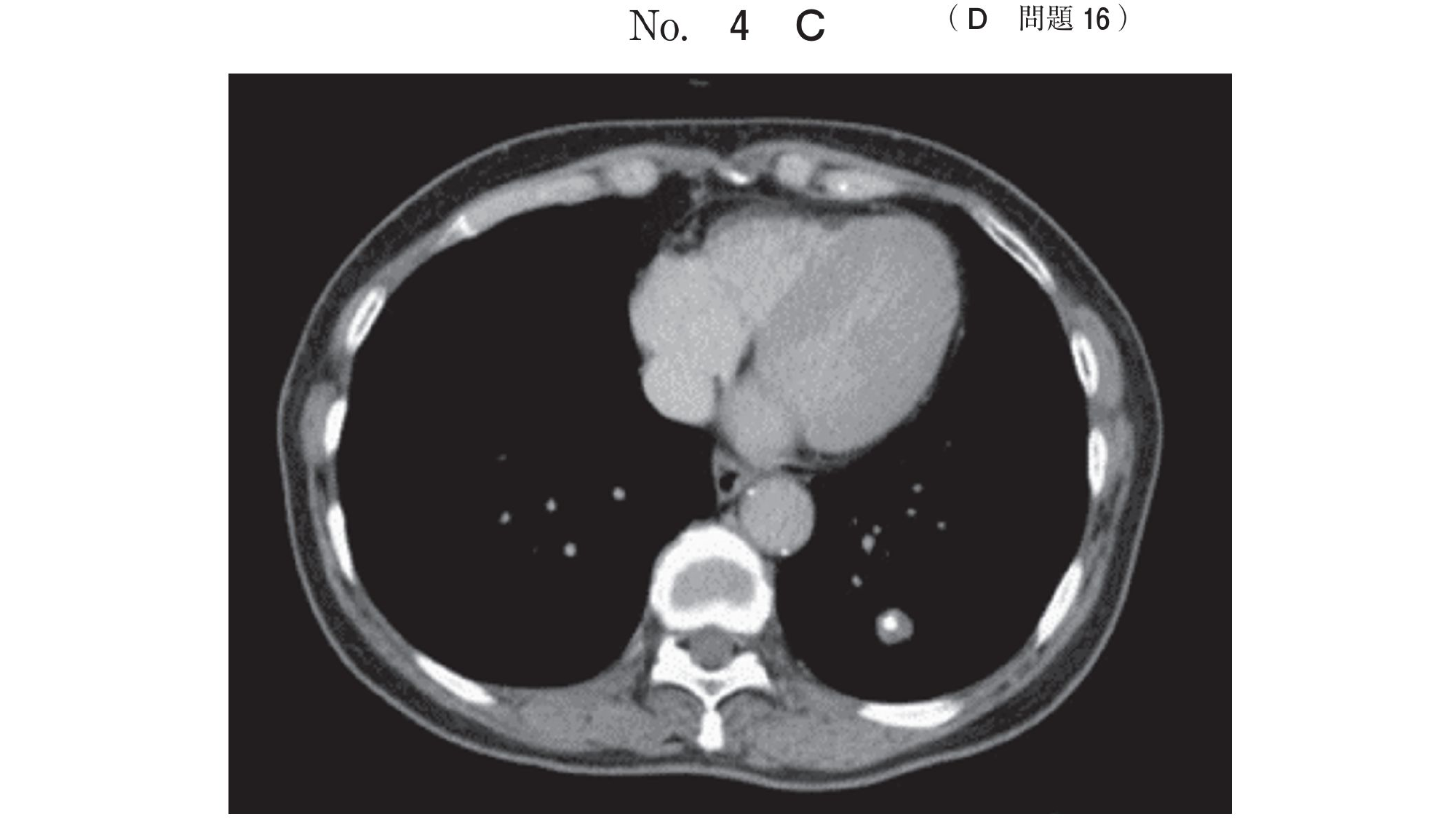

## Slide 13
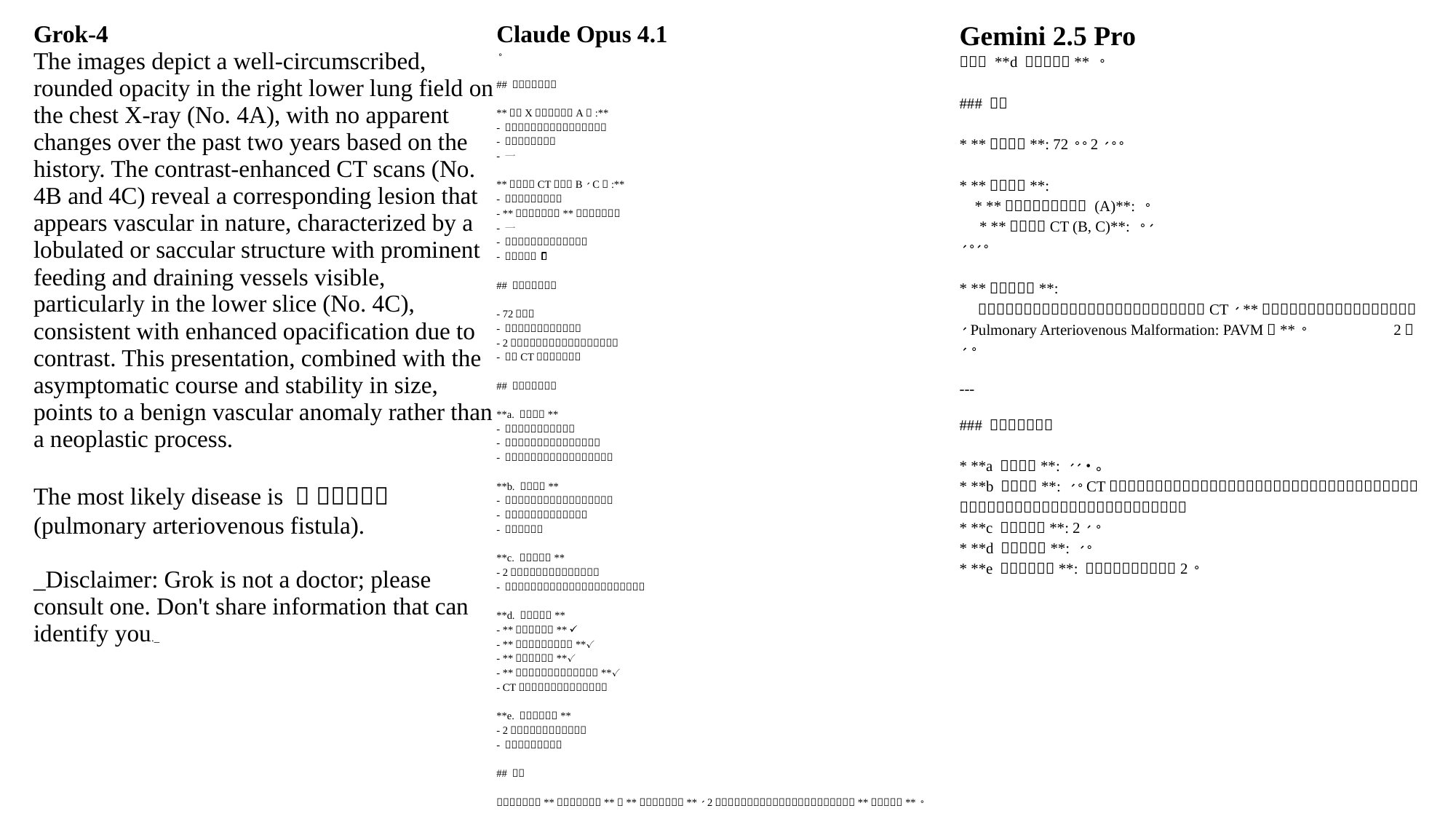

| Grok-4 The images depict a well-circumscribed, rounded opacity in the right lower lung field on the chest X-ray (No. 4A), with no apparent changes over the past two years based on the history. The contrast-enhanced CT scans (No. 4B and 4C) reveal a corresponding lesion that appears vascular in nature, characterized by a lobulated or saccular structure with prominent feeding and draining vessels visible, particularly in the lower slice (No. 4C), consistent with enhanced opacification due to contrast. This presentation, combined with the asymptomatic course and stability in size, points to a benign vascular anomaly rather than a neoplastic process. The most likely disease is ｄ 肺動静脈瘻 (pulmonary arteriovenous fistula). \_Disclaimer: Grok is not a doctor; please consult one. Don't share information that can identify you.\_ | Claude Opus 4.1 この症例を系統的に検討していきます。 ## 画像所見の分析 \*\*胸部X線写真（画像A）:\*\* - 右下肺野に類円形の結節影を認める - 境界は比較的明瞭 - 内部は均一な濃度 \*\*胸部造影CT（画像B、C）:\*\* - 右下葉に結節性病変 - \*\*造影効果が著明\*\*（重要な所見） - 内部は均一に造影される - 血管との連続性が示唆される - 流入血管・流出血管の存在が疑われる ## 臨床情報の整理 - 72歳女性 - 無症状（健診で偶然発見） - 2年間サイズ変化なし（安定している） - 造影CTで強い造影効果 ## 各選択肢の検討 \*\*a. 肺過誤腫\*\* - 通常は造影効果に乏しい - 脂肪や軟骨成分を含むことが多い - この症例の強い造影効果と合致しない \*\*b. 肺分画症\*\* - 通常は下葉に好発するが、多くは左側 - 大動脈からの異常血管を伴う - 若年者に多い \*\*c. 原発性肺癌\*\* - 2年間サイズ変化なしは非典型的 - 造影効果はあるが、これほど均一で強いことは稀 \*\*d. 肺動静脈瘻\*\* - \*\*強い造影効果\*\*（血管性病変のため）✓ - \*\*無症状のことが多い\*\*✓ - \*\*サイズが安定\*\*✓ - \*\*流入動脈と流出静脈を認める\*\*✓ - CTで血管との連続性が確認できる \*\*e. 転移性肺腫瘍\*\* - 2年間変化なしは考えにくい - 原発巣の情報がない ## 診断 画像所見（特に\*\*著明な造影効果\*\*と\*\*血管との連続性\*\*）、2年間のサイズ安定、無症状という臨床経過から、\*\*肺動静脈瘻\*\*が最も考えられます。 肺動静脈瘻は肺動脈と肺静脈の異常短絡で、造影CTでは流入動脈、瘻（nidus）、流出静脈が確認でき、強い造影効果を示すのが特徴的です。 \*\*答え：d 肺動静脈瘻\*\* | Gemini 2.5 Pro 正解は \*\*d 肺動静脈瘻\*\* です。 ### 解説 \* \*\*患者情報\*\*: 72歳の女性。健康診断で胸部異常陰影を指摘されました。2年前から同様の結節が存在し、大きさに変化はありません。自覚症状もありません。 \* \*\*画像所見\*\*: \* \*\*胸部エックス線写真 (A)\*\*: 左肺下葉に類円形の結節影が認められます。 \* \*\*胸部造影CT (B, C)\*\*: 左肺下葉に境界明瞭な結節を認めます。重要な所見として、この結節に流入する血管（肺動脈）と、流出する血管（肺静脈）が明瞭に確認できます。これは、肺の動脈と静脈が毛細血管を介さずに直接つながっていることを示しています。 \* \*\*診断の根拠\*\*: この「流入血管と流出血管を伴う結節」という特徴的なCT所見は、\*\*肺動静脈瘻（はいどうじょうみゃくろう、Pulmonary Arteriovenous Malformation: PAVM）\*\*に典型的なものです。2年間増大傾向がないという経過も、悪性腫瘍（癌）よりは良性の病変であることを支持します。 --- ### 各選択肢の評価 \* \*\*a 肺過誤腫\*\*: 良性腫瘍の一種ですが、特徴的なポップコーン様の石灰化や脂肪成分を含むことが多く、今回のような明瞭な流入・流出血管は通常みられません。 \* \*\*b 肺分画症\*\*: 正常な気管支との連続性がなく、大動脈から直接栄養血管が分岐する先天性の疾患です。CTでは体動脈からの異常な血管が同定されることが特徴であり、本症例のように肺動脈と肺静脈が直接つながる所見とは異なります。 \* \*\*c 原発性肺癌\*\*: 2年間大きさが変わらないという経過は、悪性腫瘍の可能性を著しく低くします。 \* \*\*d 肺動静脈瘻\*\*: 上記の通り、臨床経過と画像所見が最も合致する疾患です。 \* \*\*e 転移性肺腫瘍\*\*: 原発性肺癌と同様に、2年間無治療で大きさが不変であることは考えにくいです。 |
| --- | --- | --- |

## Slide 14
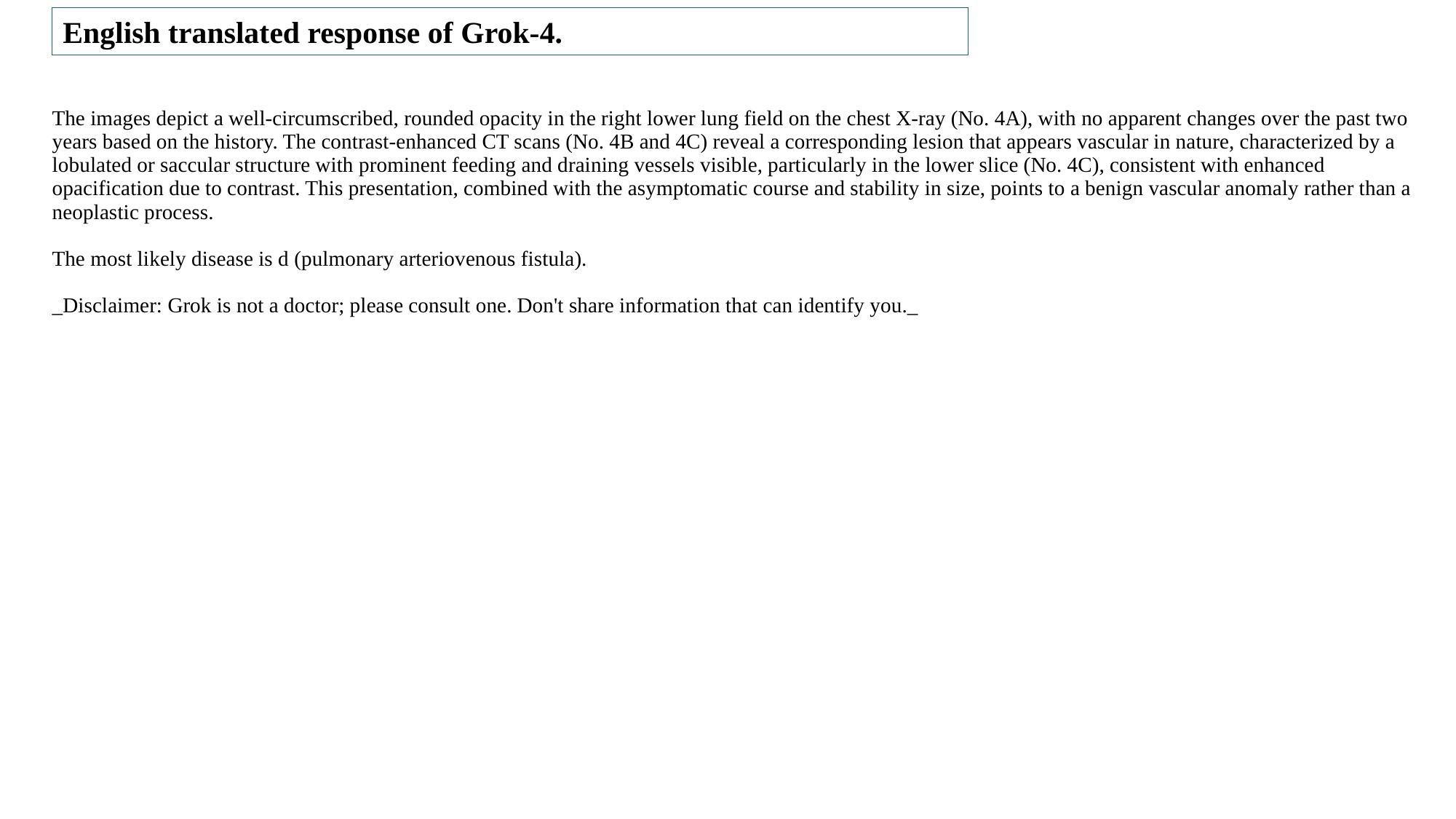

English translated response of Grok-4.
| The images depict a well-circumscribed, rounded opacity in the right lower lung field on the chest X-ray (No. 4A), with no apparent changes over the past two years based on the history. The contrast-enhanced CT scans (No. 4B and 4C) reveal a corresponding lesion that appears vascular in nature, characterized by a lobulated or saccular structure with prominent feeding and draining vessels visible, particularly in the lower slice (No. 4C), consistent with enhanced opacification due to contrast. This presentation, combined with the asymptomatic course and stability in size, points to a benign vascular anomaly rather than a neoplastic process. The most likely disease is d (pulmonary arteriovenous fistula). \_Disclaimer: Grok is not a doctor; please consult one. Don't share information that can identify you.\_ |
| --- |

## Slide 15
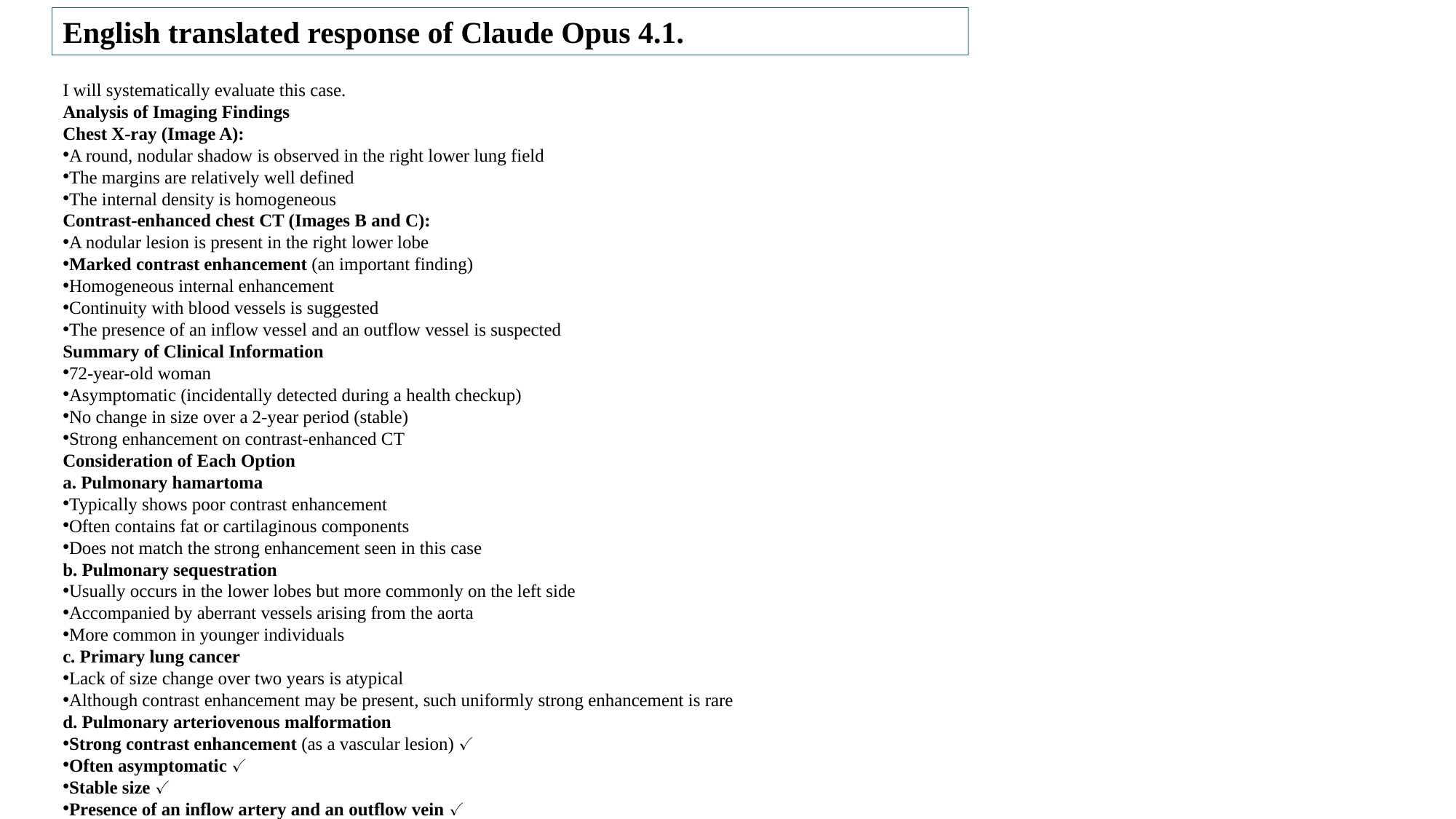

English translated response of Claude Opus 4.1.
I will systematically evaluate this case.
Analysis of Imaging Findings
Chest X-ray (Image A):
A round, nodular shadow is observed in the right lower lung field
The margins are relatively well defined
The internal density is homogeneous
Contrast-enhanced chest CT (Images B and C):
A nodular lesion is present in the right lower lobe
Marked contrast enhancement (an important finding)
Homogeneous internal enhancement
Continuity with blood vessels is suggested
The presence of an inflow vessel and an outflow vessel is suspected
Summary of Clinical Information
72-year-old woman
Asymptomatic (incidentally detected during a health checkup)
No change in size over a 2-year period (stable)
Strong enhancement on contrast-enhanced CT
Consideration of Each Option
a. Pulmonary hamartoma
Typically shows poor contrast enhancement
Often contains fat or cartilaginous components
Does not match the strong enhancement seen in this case
b. Pulmonary sequestration
Usually occurs in the lower lobes but more commonly on the left side
Accompanied by aberrant vessels arising from the aorta
More common in younger individuals
c. Primary lung cancer
Lack of size change over two years is atypical
Although contrast enhancement may be present, such uniformly strong enhancement is rare
d. Pulmonary arteriovenous malformation
Strong contrast enhancement (as a vascular lesion) ✓
Often asymptomatic ✓
Stable size ✓
Presence of an inflow artery and an outflow vein ✓
Continuity with blood vessels can be confirmed on CT
e. Metastatic lung tumor
Unlikely to remain unchanged over two years
No information regarding a primary lesion
Diagnosis
Based on the imaging findings—particularly the marked contrast enhancement and continuity with blood vessels—as well as the stable size over two years and asymptomatic clinical course, pulmonary arteriovenous malformation is the most likely diagnosis.
Pulmonary arteriovenous malformation is an abnormal shunt between the pulmonary artery and pulmonary vein; on contrast-enhanced CT, the inflow artery, nidus, and outflow vein can be identified, and strong contrast enhancement is a characteristic feature.
Answer: d. Pulmonary arteriovenous malformation

## Slide 16
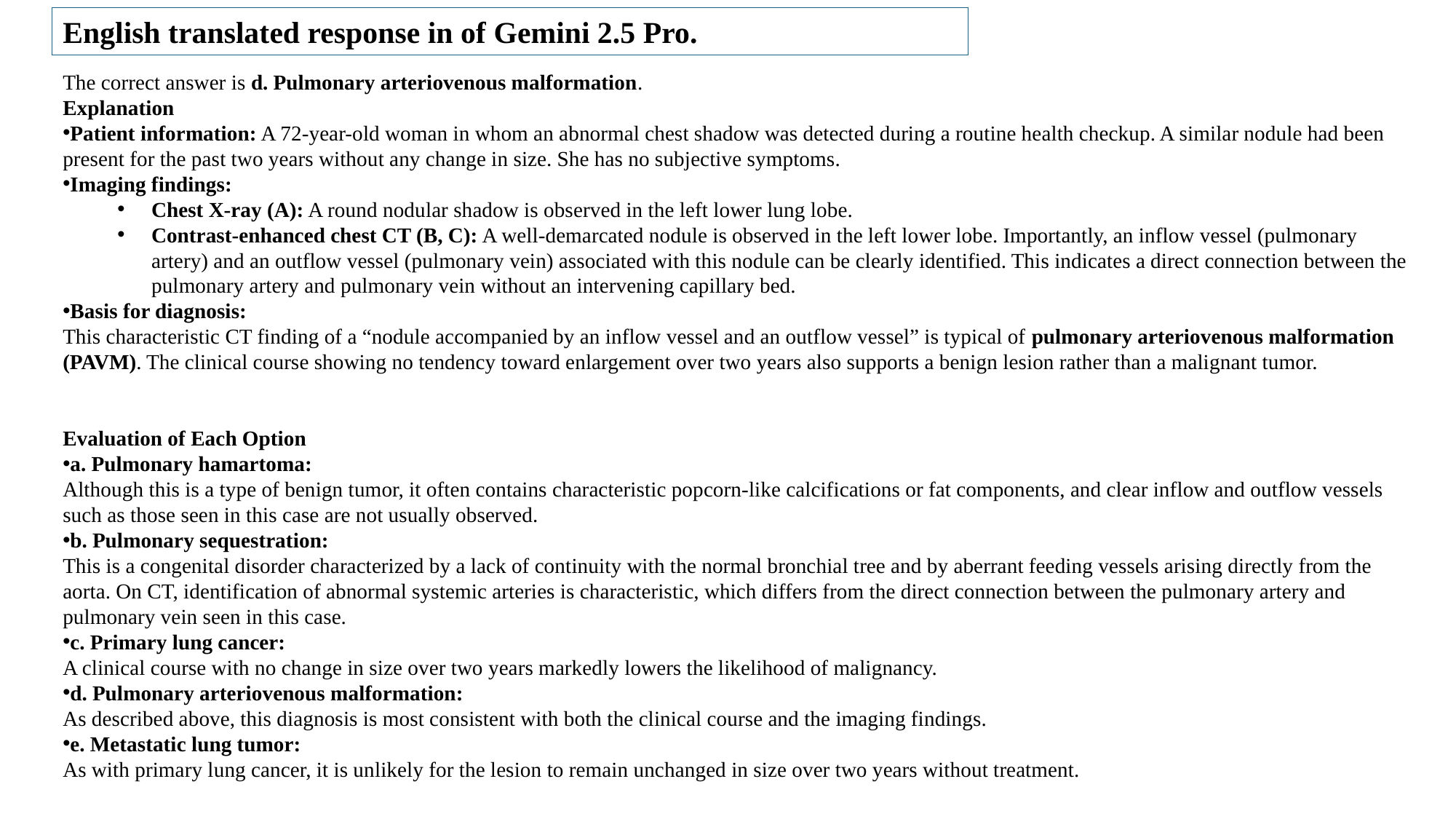

English translated response in of Gemini 2.5 Pro.
The correct answer is d. Pulmonary arteriovenous malformation.
Explanation
Patient information: A 72-year-old woman in whom an abnormal chest shadow was detected during a routine health checkup. A similar nodule had been present for the past two years without any change in size. She has no subjective symptoms.
Imaging findings:
Chest X-ray (A): A round nodular shadow is observed in the left lower lung lobe.
Contrast-enhanced chest CT (B, C): A well-demarcated nodule is observed in the left lower lobe. Importantly, an inflow vessel (pulmonary artery) and an outflow vessel (pulmonary vein) associated with this nodule can be clearly identified. This indicates a direct connection between the pulmonary artery and pulmonary vein without an intervening capillary bed.
Basis for diagnosis:
This characteristic CT finding of a “nodule accompanied by an inflow vessel and an outflow vessel” is typical of pulmonary arteriovenous malformation (PAVM). The clinical course showing no tendency toward enlargement over two years also supports a benign lesion rather than a malignant tumor.
Evaluation of Each Option
a. Pulmonary hamartoma:
Although this is a type of benign tumor, it often contains characteristic popcorn-like calcifications or fat components, and clear inflow and outflow vessels such as those seen in this case are not usually observed.
b. Pulmonary sequestration:
This is a congenital disorder characterized by a lack of continuity with the normal bronchial tree and by aberrant feeding vessels arising directly from the aorta. On CT, identification of abnormal systemic arteries is characteristic, which differs from the direct connection between the pulmonary artery and pulmonary vein seen in this case.
c. Primary lung cancer:
A clinical course with no change in size over two years markedly lowers the likelihood of malignancy.
d. Pulmonary arteriovenous malformation:
As described above, this diagnosis is most consistent with both the clinical course and the imaging findings.
e. Metastatic lung tumor:
As with primary lung cancer, it is unlikely for the lesion to remain unchanged in size over two years without treatment.

## Slide 17
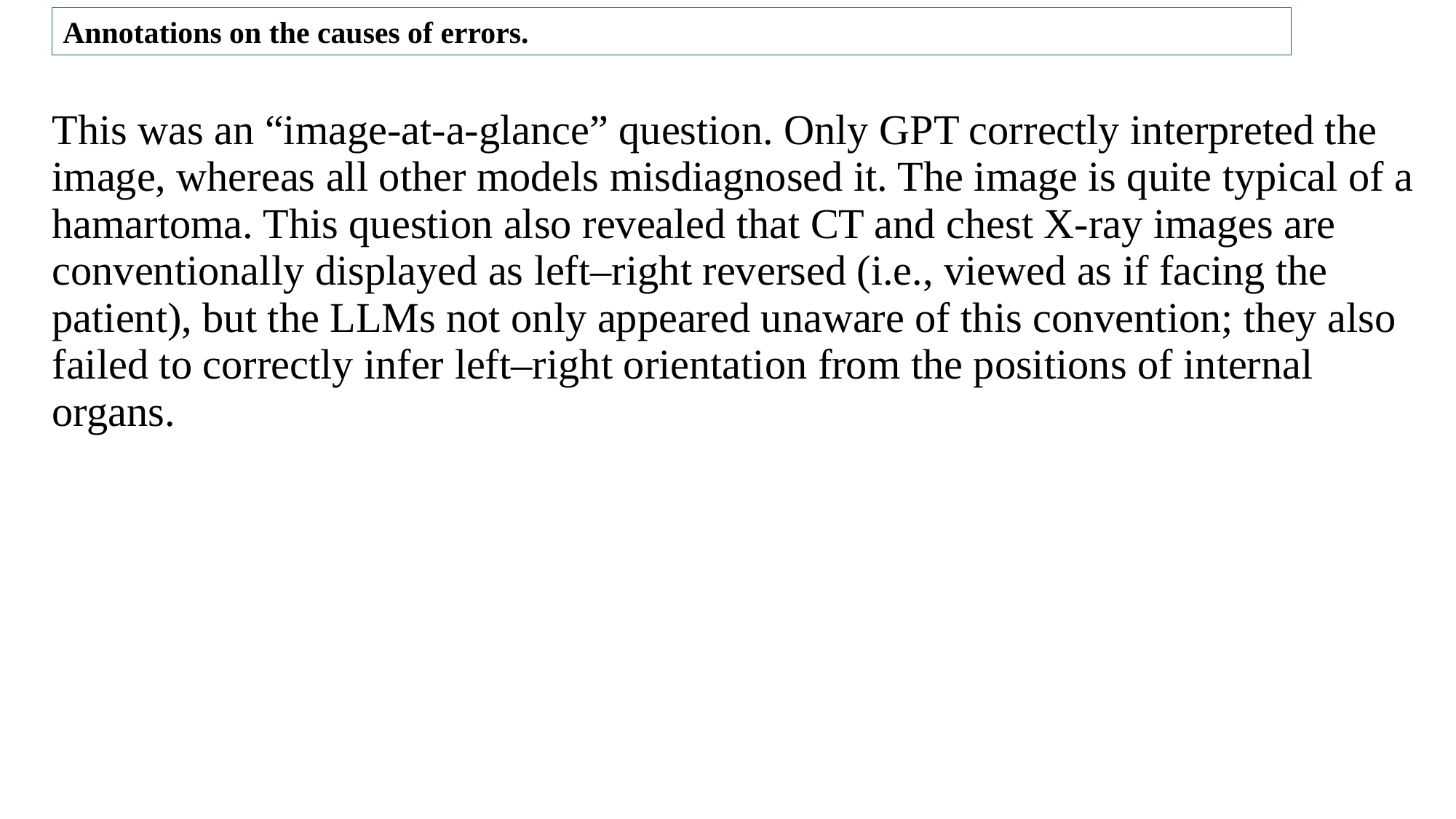

Annotations on the causes of errors.
| This was an “image-at-a-glance” question. Only GPT correctly interpreted the image, whereas all other models misdiagnosed it. The image is quite typical of a hamartoma. This question also revealed that CT and chest X-ray images are conventionally displayed as left–right reversed (i.e., viewed as if facing the patient), but the LLMs not only appeared unaware of this convention; they also failed to correctly infer left–right orientation from the positions of internal organs. |
| --- |

## Slide 18
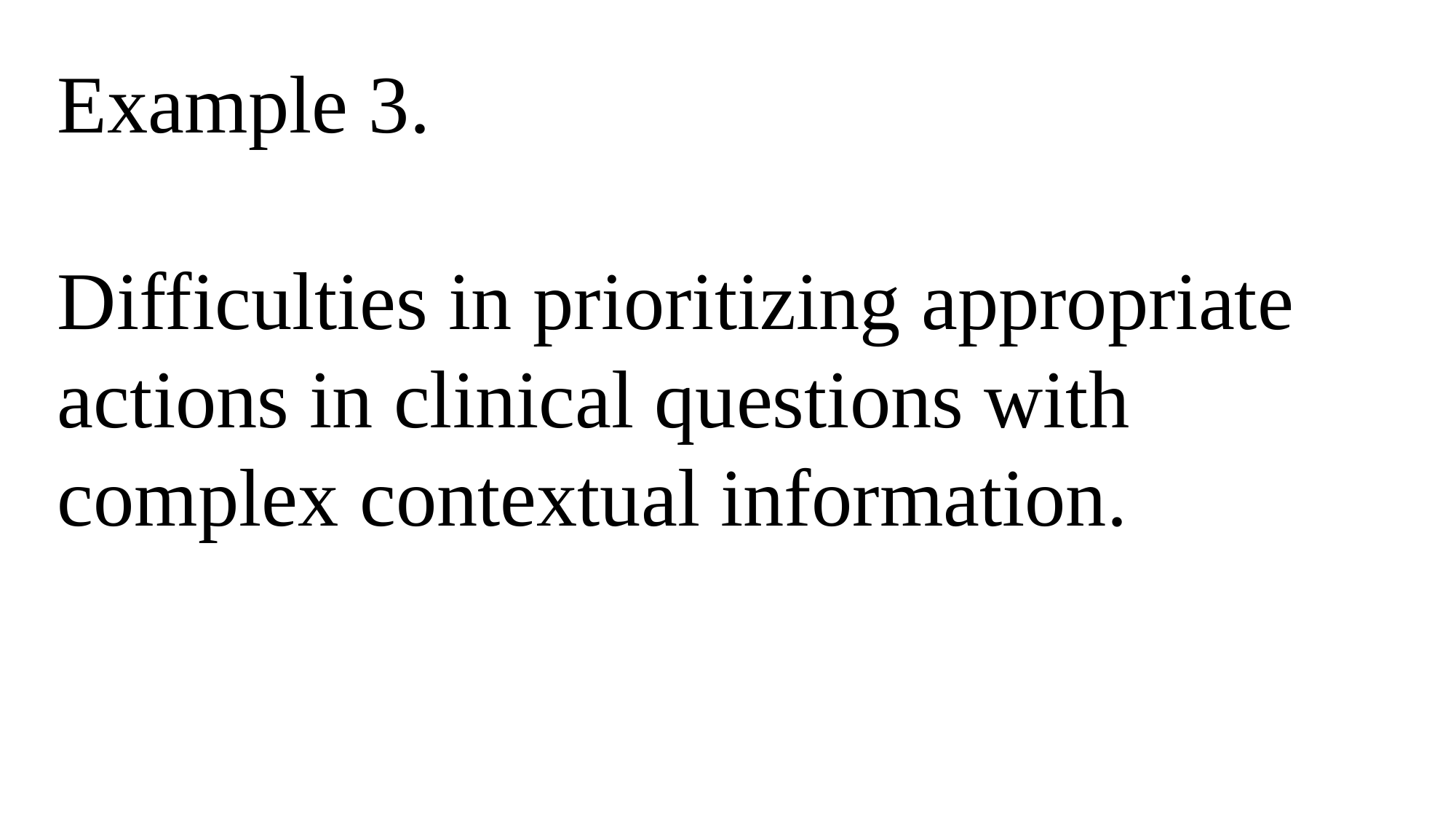

Example 3.
Difficulties in prioritizing appropriate actions in clinical questions with complex contextual information.

## Slide 19
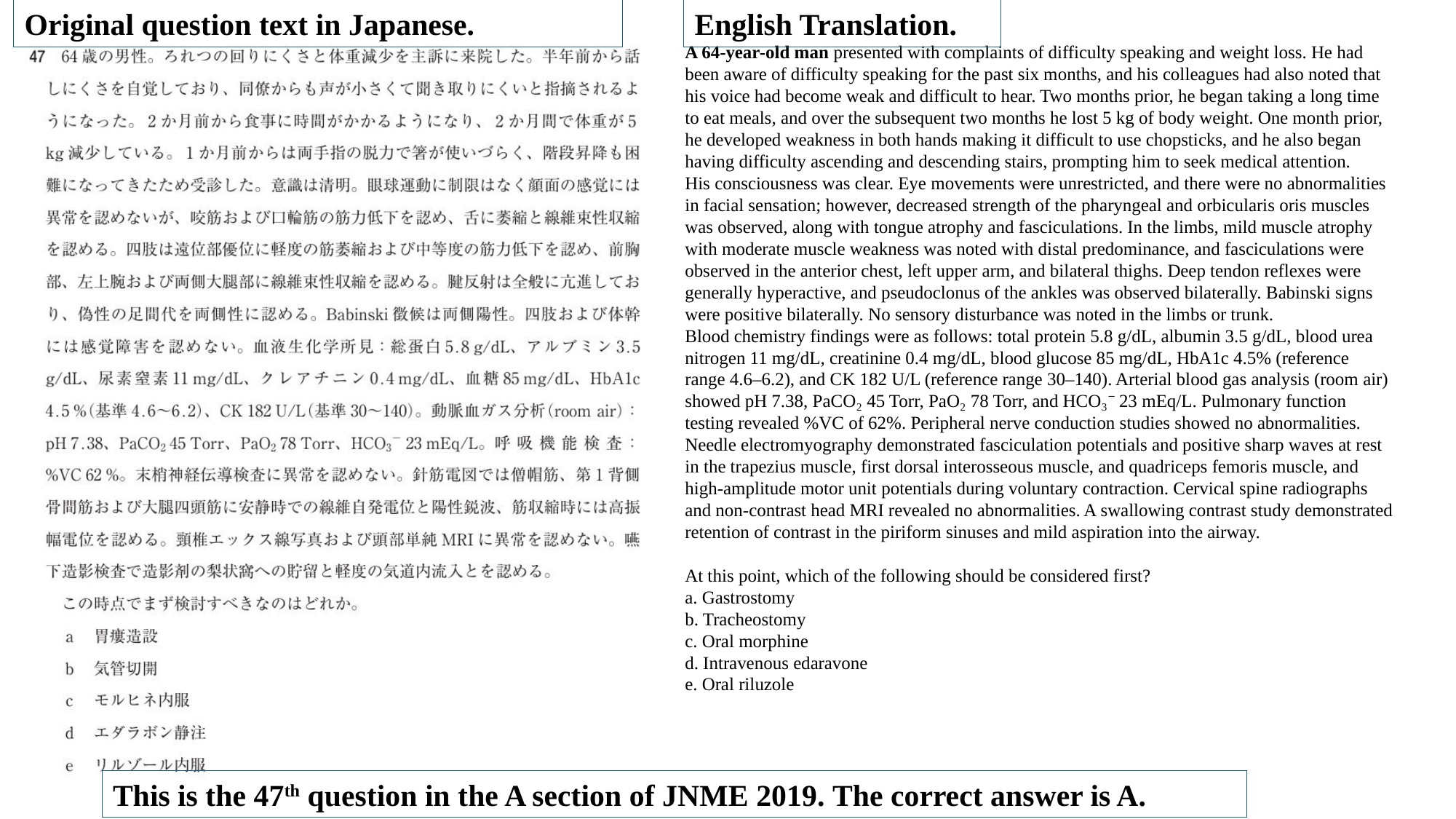

Original question text in Japanese.
English Translation.
A 64-year-old man presented with complaints of difficulty speaking and weight loss. He had been aware of difficulty speaking for the past six months, and his colleagues had also noted that his voice had become weak and difficult to hear. Two months prior, he began taking a long time to eat meals, and over the subsequent two months he lost 5 kg of body weight. One month prior, he developed weakness in both hands making it difficult to use chopsticks, and he also began having difficulty ascending and descending stairs, prompting him to seek medical attention.
His consciousness was clear. Eye movements were unrestricted, and there were no abnormalities in facial sensation; however, decreased strength of the pharyngeal and orbicularis oris muscles was observed, along with tongue atrophy and fasciculations. In the limbs, mild muscle atrophy with moderate muscle weakness was noted with distal predominance, and fasciculations were observed in the anterior chest, left upper arm, and bilateral thighs. Deep tendon reflexes were generally hyperactive, and pseudoclonus of the ankles was observed bilaterally. Babinski signs were positive bilaterally. No sensory disturbance was noted in the limbs or trunk.
Blood chemistry findings were as follows: total protein 5.8 g/dL, albumin 3.5 g/dL, blood urea nitrogen 11 mg/dL, creatinine 0.4 mg/dL, blood glucose 85 mg/dL, HbA1c 4.5% (reference range 4.6–6.2), and CK 182 U/L (reference range 30–140). Arterial blood gas analysis (room air) showed pH 7.38, PaCO₂ 45 Torr, PaO₂ 78 Torr, and HCO₃⁻ 23 mEq/L. Pulmonary function testing revealed %VC of 62%. Peripheral nerve conduction studies showed no abnormalities. Needle electromyography demonstrated fasciculation potentials and positive sharp waves at rest in the trapezius muscle, first dorsal interosseous muscle, and quadriceps femoris muscle, and high-amplitude motor unit potentials during voluntary contraction. Cervical spine radiographs and non-contrast head MRI revealed no abnormalities. A swallowing contrast study demonstrated retention of contrast in the piriform sinuses and mild aspiration into the airway.
At this point, which of the following should be considered first?
a. Gastrostomy
b. Tracheostomy
c. Oral morphine
d. Intravenous edaravone
e. Oral riluzole
This is the 47th question in the A section of JNME 2019. The correct answer is A.

## Slide 20
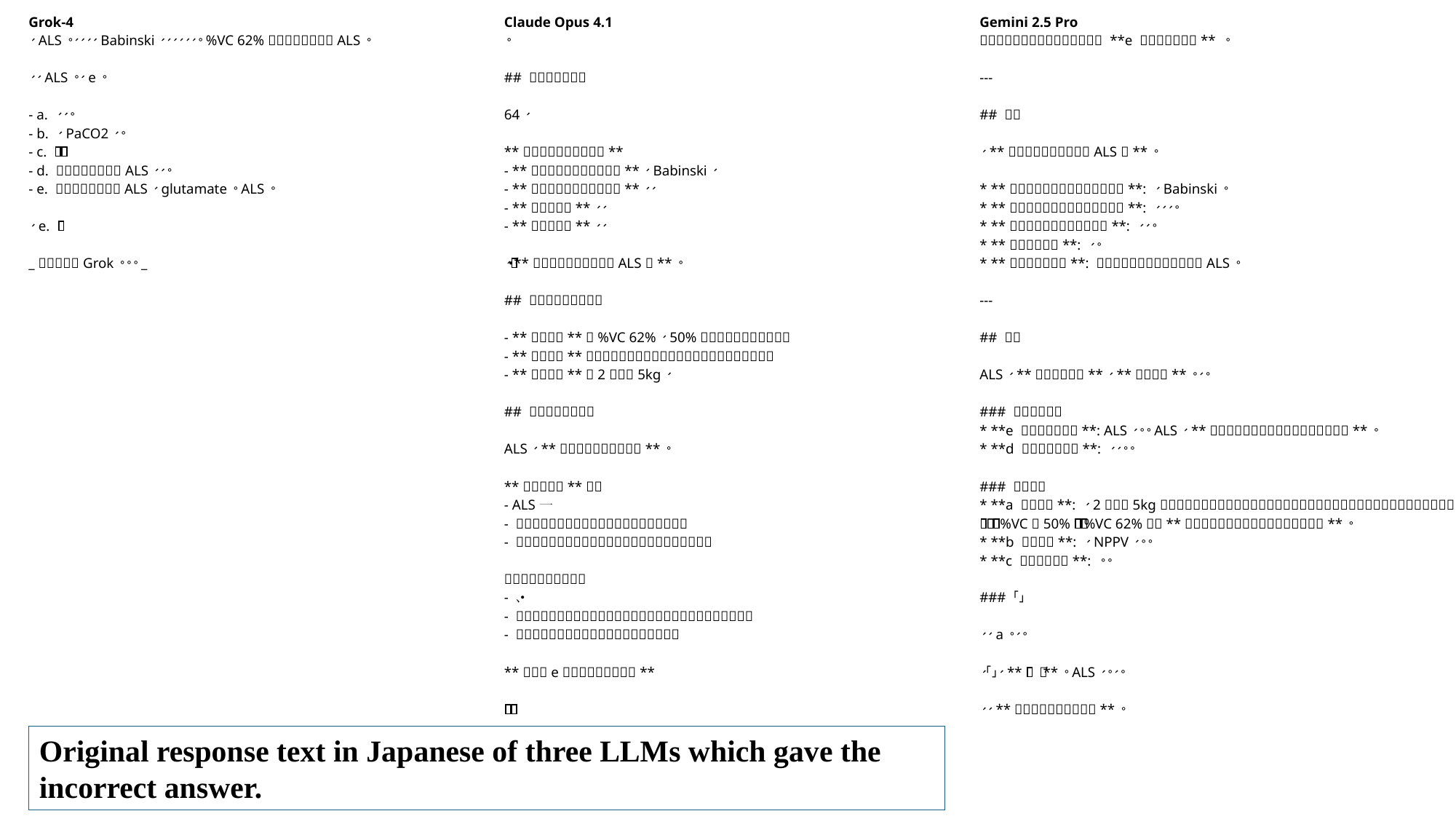

| Grok-4 この患者の症状と検査所見から、筋萎縮性側索硬化症（ALS）が強く疑われる。球麻痺（舌萎縮、構音障害、嚥下障害）、上位運動ニューロン徴候（腱反射亢進、Babinski徴候陽性）、下位運動ニューロン徴候（筋萎縮、線維束性収縮）、針筋電図の神経原性変化、末梢神経伝導正常、画像異常なし、という所見が典型的に一致する。呼吸機能低下（%VC 62%）と誤嚥の兆候もALSの進行を示唆する。 この時点でまず検討すべきは、疾患修飾療法の開始であり、ALSの進行を遅らせる可能性のある治療薬を優先する。選択肢のうち、以下の理由でeが適切である。 - a. 胃瘻造設：嚥下障害と体重減少があるため将来的に検討されるが、誤嚥は軽度で即時必要とは言えず、まず疾患本体への介入を優先。 - b. 気管切開：呼吸機能低下はあるが、PaCO2正常で重度の呼吸不全に至っていないため、即時適応ではない。 - c. モルヒネ内服：疼痛や呼吸苦の緩和に用いるが、本症例に該当する症状はない。 - d. エダラボン静注：ALSの治療薬として日本で承認されているが、通常リルゾールに次いで追加検討されることが多く、静注のため負担が大きい。 - e. リルゾール内服：ALSの標準的な疾患修飾療法として第一に検討され、glutamate神経毒性を抑制し生存期間を延長する可能性がある。日本ALS診療ガイドラインでも診断後早期の投与が推奨される。 したがって、答えはe. リルゾール内服。 \_免責事項：Grokは医師ではありません。医師に相談してください。あなたを特定できる情報を共有しないでください。\_ | Claude Opus 4.1 この症例を分析させていただきます。 ## 症例のポイント 64歳男性で、以下の特徴的な所見があります： \*\*神経学的所見の整理：\*\* - \*\*上位運動ニューロン徴候\*\*：腱反射亢進、Babinski徴候陽性、偽性足間代 - \*\*下位運動ニューロン徴候\*\*：筋萎縮、線維束性収縮、舌萎縮 - \*\*球麻痺症状\*\*：構音障害、嚥下障害、咬筋・口輪筋の筋力低下 - \*\*筋電図所見\*\*：神経原性変化（線維自発電位、陽性鋭波、高振幅電位） これらの所見は、上位・下位運動ニューロンの両方が障害される\*\*筋萎縮性側索硬化症（ALS）\*\*を強く示唆します。 ## 現時点での状態評価 - \*\*呼吸機能\*\*：%VC 62%（軽度低下だが、気管切開の適応となる50%以下には至っていない） - \*\*嚥下機能\*\*：軽度の誤嚥はあるが、まだ経口摂取可能なレベル - \*\*体重減少\*\*：2か月で5kgと進行性だが、緊急の栄養管理介入が必要な状態ではない ## 治療選択の考え方 ALSと診断された時点で、\*\*疾患修飾薬の早期導入\*\*が最優先となります。 \*\*リルゾール\*\*は： - ALSで唯一生存期間延長効果が証明されている薬剤 - グルタミン酸拮抗作用により神経細胞死を抑制 - 診断後早期に開始することで最大の効果が期待できる 他の選択肢について： - 胃瘻造設・気管切開：将来的には必要となる可能性があるが、現時点では時期尚早 - エダラボン：進行抑制効果はあるが、リルゾールの方が第一選択 - モルヒネ：呼吸苦がない現時点では適応なし \*\*答え：e（リルゾール内服）\*\* まずリルゾールによる治療を開始し、症状の進行に応じて胃瘻造設や呼吸管理について検討していくことが標準的な治療方針となります。 | Gemini 2.5 Pro この症例でまず検討すべきなのは \*\*e リルゾール内服\*\* です。 --- ## 診断 この患者さんは、以下の点から\*\*筋萎縮性側索硬化症（ALS）\*\*が強く疑われます。 \* \*\*上位運動ニューロン障害の兆候\*\*: 腱反射の亢進、病的反射（Babinski徴候）陽性。 \* \*\*下位運動ニューロン障害の兆候\*\*: 筋萎縮、筋力低下、舌や全身の線維束性収縮、針筋電図での脱神経所見。 \* \*\*球麻痺（延髄麻痺）の症状\*\*: ろれつの回りにくさ（構音障害）、食事に時間がかかる（嚥下障害）、舌の萎縮。 \* \*\*進行性の経過\*\*: 半年前から症状が出現し、徐々に悪化している。 \* \*\*感覚障害がない\*\*: 感覚神経は障害されないのがALSの特徴です。 --- ## 解説 ALSの治療は、疾患の進行を遅らせる\*\*疾患修飾療法\*\*と、症状を和らげる\*\*対症療法\*\*の二本柱で行われます。提示された選択肢について、以下のように考えられます。 ### 疾患修飾療法 \* \*\*e リルゾール内服\*\*: ALSの進行を遅らせ、生存期間を延長する効果が科学的に証明されている標準的な治療薬です。グルタミン酸の神経毒性を抑制する作用があります。ALSと診断された場合、\*\*可能な限り早期に開始することが推奨\*\*されています。 \* \*\*d エダラボン静注\*\*: 日本で開発された治療薬で、フリーラジカルを除去することで神経細胞を保護し、病気の進行を抑制する効果があります。リルゾールと並行して用いられることもあります。 ### 対症療法 \* \*\*a 胃瘻造設\*\*: 嚥下障害が進行し、体重減少（2ヶ月で5kg）や誤嚥（嚥下造影で確認）が見られるため、栄養補給と誤嚥性肺炎の予防のために非常に重要な治療です。特に、呼吸機能が低下する前（一般的に努力性肺活量 %VCが50%を切る前）に行うことが望ましいため、この患者さん（%VC 62%）は\*\*胃瘻造設を検討すべき良いタイミング\*\*です。 \* \*\*b 気管切開\*\*: 呼吸筋の麻痺がさらに進行し、非侵襲的陽圧換気療法（NPPV、マスク式の人工呼吸器）でも呼吸管理が困難になった場合に検討されます。現時点ではまだ早計です。 \* \*\*c モルヒネ内服\*\*: 主に病気の末期における呼吸困難感の緩和などに用いられます。現時点では適応がありません。 ### なぜ「リルゾール内服」をまず検討するのか？ この患者さんは、栄養障害や呼吸機能の低下が始まっており、胃瘻造設（選択肢a）の検討も急務です。臨床現場では、リルゾールの開始と胃瘻の検討はほぼ同時に進められます。 しかし、「治療としてまず何をすべきか」と問われた場合、疾患そのものの進行に働きかける\*\*疾患修飾薬（リルゾール）の開始が最も基本的な第一歩\*\*となります。リルゾールはALSの治療の根幹をなすものであり、その効果を最大限に得るためには早期からの介入が重要だからです。胃瘻造設などの対症療法は、病状の進行に応じて適切なタイミングで導入していく治療と位置づけられます。 したがって、診断が確定したこの時点で、まず検討すべきは標準的疾患修飾薬である\*\*リルゾールの内服開始\*\*となります。 |
| --- | --- | --- |
Original response text in Japanese of three LLMs which gave the incorrect answer.

## Slide 21
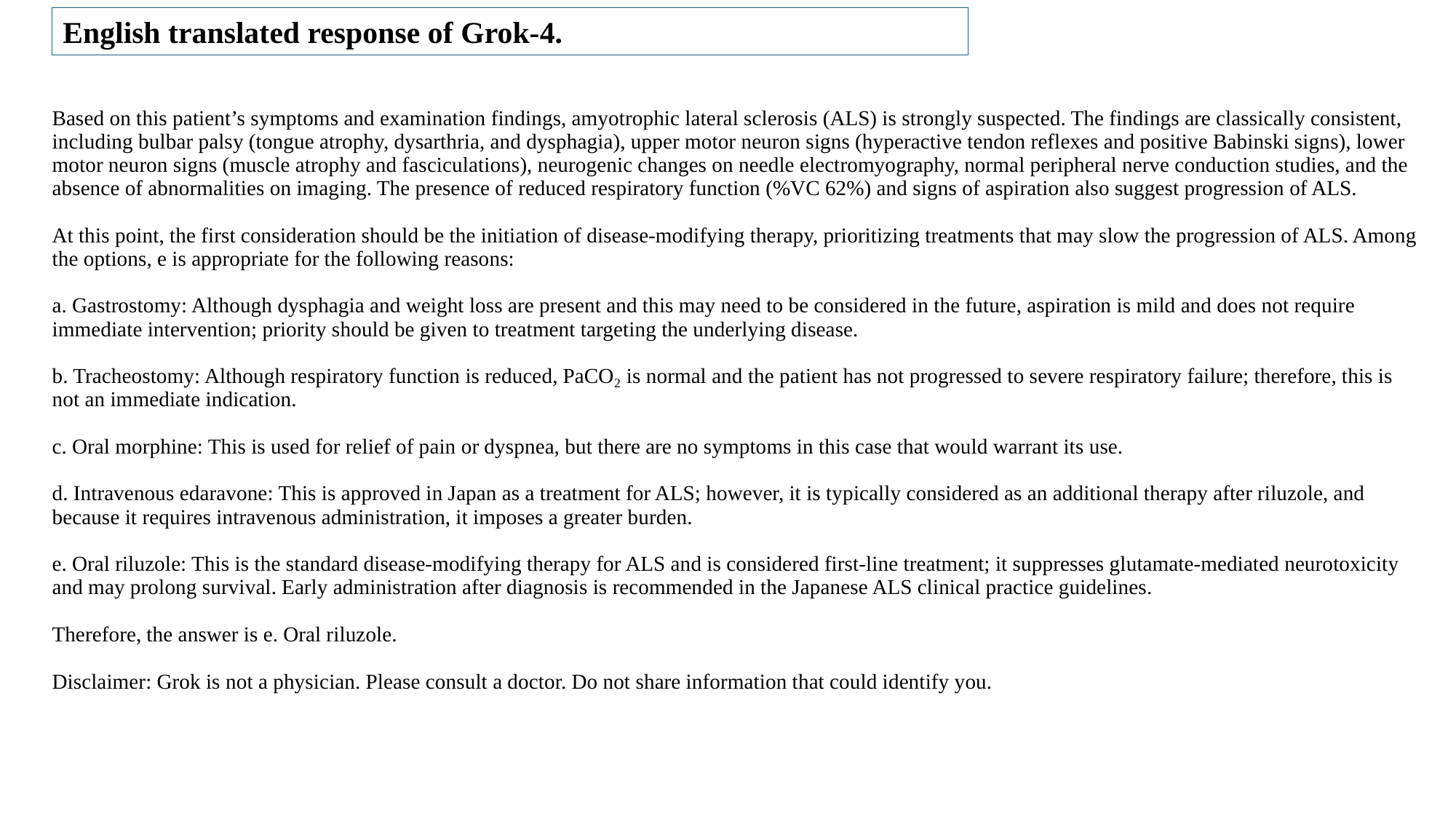

English translated response of Grok-4.
| Based on this patient’s symptoms and examination findings, amyotrophic lateral sclerosis (ALS) is strongly suspected. The findings are classically consistent, including bulbar palsy (tongue atrophy, dysarthria, and dysphagia), upper motor neuron signs (hyperactive tendon reflexes and positive Babinski signs), lower motor neuron signs (muscle atrophy and fasciculations), neurogenic changes on needle electromyography, normal peripheral nerve conduction studies, and the absence of abnormalities on imaging. The presence of reduced respiratory function (%VC 62%) and signs of aspiration also suggest progression of ALS. At this point, the first consideration should be the initiation of disease-modifying therapy, prioritizing treatments that may slow the progression of ALS. Among the options, e is appropriate for the following reasons: a. Gastrostomy: Although dysphagia and weight loss are present and this may need to be considered in the future, aspiration is mild and does not require immediate intervention; priority should be given to treatment targeting the underlying disease. b. Tracheostomy: Although respiratory function is reduced, PaCO₂ is normal and the patient has not progressed to severe respiratory failure; therefore, this is not an immediate indication. c. Oral morphine: This is used for relief of pain or dyspnea, but there are no symptoms in this case that would warrant its use. d. Intravenous edaravone: This is approved in Japan as a treatment for ALS; however, it is typically considered as an additional therapy after riluzole, and because it requires intravenous administration, it imposes a greater burden. e. Oral riluzole: This is the standard disease-modifying therapy for ALS and is considered first-line treatment; it suppresses glutamate-mediated neurotoxicity and may prolong survival. Early administration after diagnosis is recommended in the Japanese ALS clinical practice guidelines. Therefore, the answer is e. Oral riluzole. Disclaimer: Grok is not a physician. Please consult a doctor. Do not share information that could identify you. |
| --- |

## Slide 22
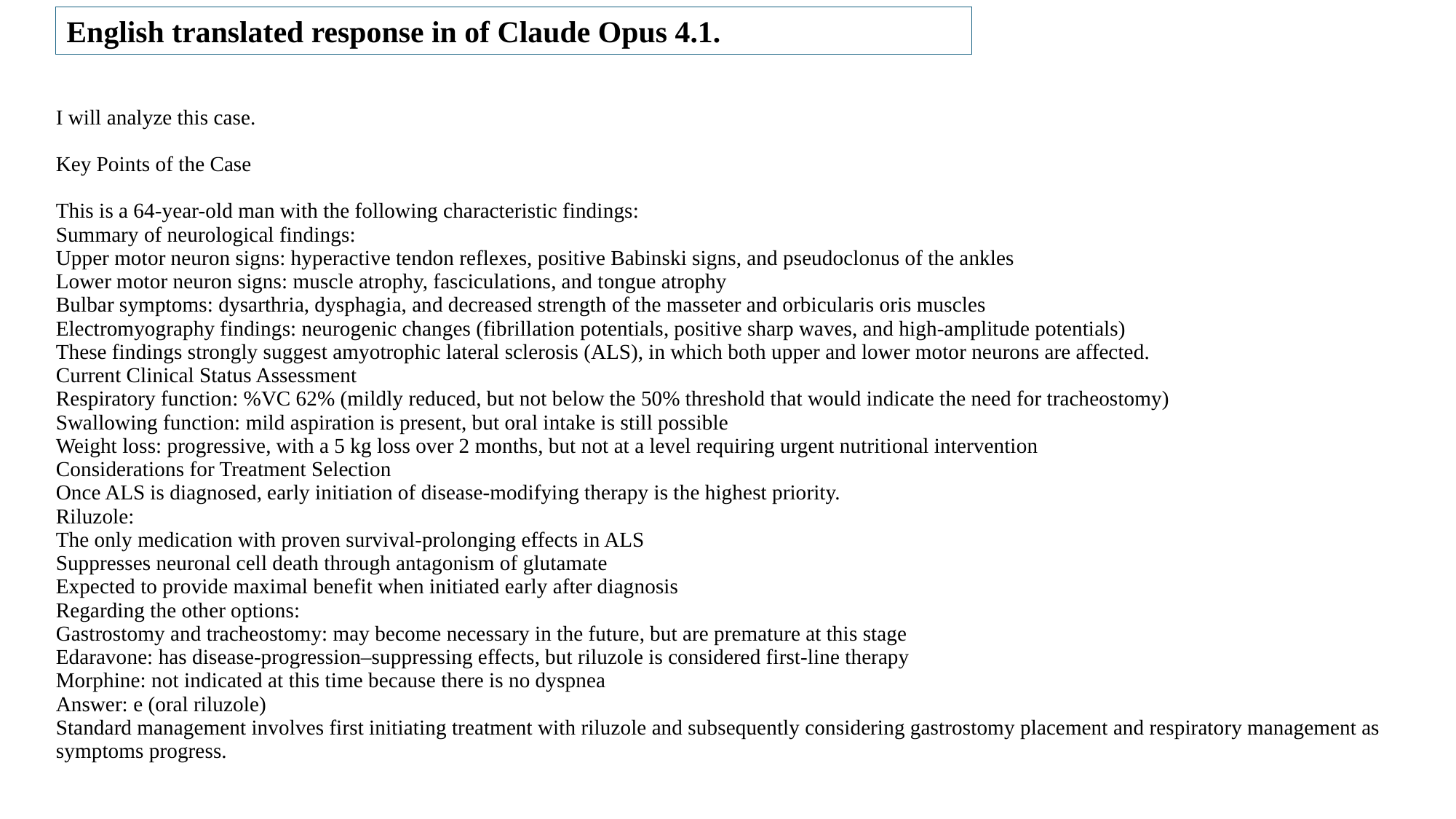

English translated response in of Claude Opus 4.1.
| I will analyze this case. Key Points of the Case This is a 64-year-old man with the following characteristic findings: Summary of neurological findings: Upper motor neuron signs: hyperactive tendon reflexes, positive Babinski signs, and pseudoclonus of the ankles Lower motor neuron signs: muscle atrophy, fasciculations, and tongue atrophy Bulbar symptoms: dysarthria, dysphagia, and decreased strength of the masseter and orbicularis oris muscles Electromyography findings: neurogenic changes (fibrillation potentials, positive sharp waves, and high-amplitude potentials) These findings strongly suggest amyotrophic lateral sclerosis (ALS), in which both upper and lower motor neurons are affected. Current Clinical Status Assessment Respiratory function: %VC 62% (mildly reduced, but not below the 50% threshold that would indicate the need for tracheostomy) Swallowing function: mild aspiration is present, but oral intake is still possible Weight loss: progressive, with a 5 kg loss over 2 months, but not at a level requiring urgent nutritional intervention Considerations for Treatment Selection Once ALS is diagnosed, early initiation of disease-modifying therapy is the highest priority. Riluzole: The only medication with proven survival-prolonging effects in ALS Suppresses neuronal cell death through antagonism of glutamate Expected to provide maximal benefit when initiated early after diagnosis Regarding the other options: Gastrostomy and tracheostomy: may become necessary in the future, but are premature at this stage Edaravone: has disease-progression–suppressing effects, but riluzole is considered first-line therapy Morphine: not indicated at this time because there is no dyspnea Answer: e (oral riluzole) Standard management involves first initiating treatment with riluzole and subsequently considering gastrostomy placement and respiratory management as symptoms progress. |
| --- |

## Slide 23
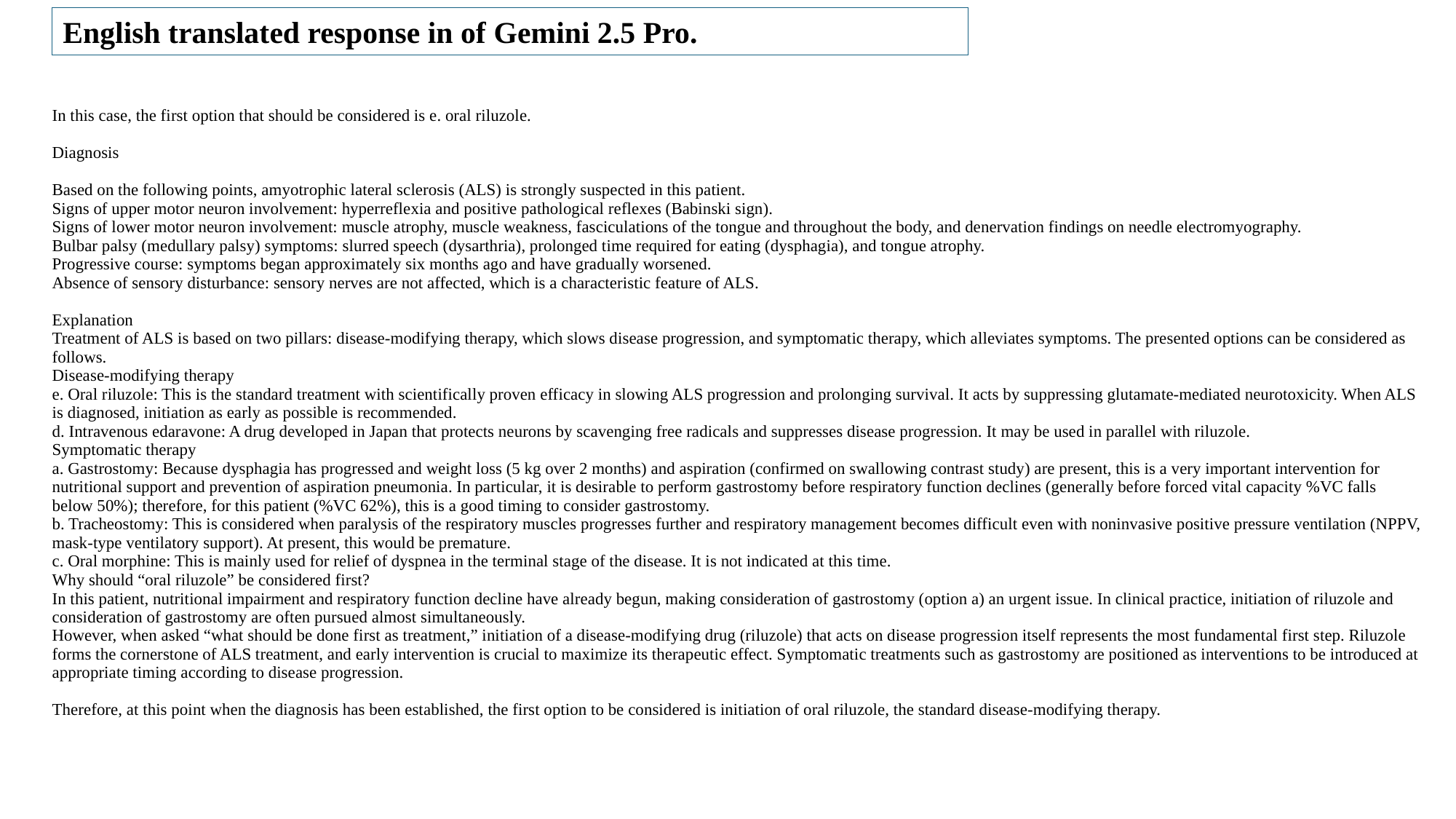

English translated response in of Gemini 2.5 Pro.
| In this case, the first option that should be considered is e. oral riluzole. Diagnosis Based on the following points, amyotrophic lateral sclerosis (ALS) is strongly suspected in this patient. Signs of upper motor neuron involvement: hyperreflexia and positive pathological reflexes (Babinski sign). Signs of lower motor neuron involvement: muscle atrophy, muscle weakness, fasciculations of the tongue and throughout the body, and denervation findings on needle electromyography. Bulbar palsy (medullary palsy) symptoms: slurred speech (dysarthria), prolonged time required for eating (dysphagia), and tongue atrophy. Progressive course: symptoms began approximately six months ago and have gradually worsened. Absence of sensory disturbance: sensory nerves are not affected, which is a characteristic feature of ALS. Explanation Treatment of ALS is based on two pillars: disease-modifying therapy, which slows disease progression, and symptomatic therapy, which alleviates symptoms. The presented options can be considered as follows. Disease-modifying therapy e. Oral riluzole: This is the standard treatment with scientifically proven efficacy in slowing ALS progression and prolonging survival. It acts by suppressing glutamate-mediated neurotoxicity. When ALS is diagnosed, initiation as early as possible is recommended. d. Intravenous edaravone: A drug developed in Japan that protects neurons by scavenging free radicals and suppresses disease progression. It may be used in parallel with riluzole. Symptomatic therapy a. Gastrostomy: Because dysphagia has progressed and weight loss (5 kg over 2 months) and aspiration (confirmed on swallowing contrast study) are present, this is a very important intervention for nutritional support and prevention of aspiration pneumonia. In particular, it is desirable to perform gastrostomy before respiratory function declines (generally before forced vital capacity %VC falls below 50%); therefore, for this patient (%VC 62%), this is a good timing to consider gastrostomy. b. Tracheostomy: This is considered when paralysis of the respiratory muscles progresses further and respiratory management becomes difficult even with noninvasive positive pressure ventilation (NPPV, mask-type ventilatory support). At present, this would be premature. c. Oral morphine: This is mainly used for relief of dyspnea in the terminal stage of the disease. It is not indicated at this time. Why should “oral riluzole” be considered first? In this patient, nutritional impairment and respiratory function decline have already begun, making consideration of gastrostomy (option a) an urgent issue. In clinical practice, initiation of riluzole and consideration of gastrostomy are often pursued almost simultaneously. However, when asked “what should be done first as treatment,” initiation of a disease-modifying drug (riluzole) that acts on disease progression itself represents the most fundamental first step. Riluzole forms the cornerstone of ALS treatment, and early intervention is crucial to maximize its therapeutic effect. Symptomatic treatments such as gastrostomy are positioned as interventions to be introduced at appropriate timing according to disease progression. Therefore, at this point when the diagnosis has been established, the first option to be considered is initiation of oral riluzole, the standard disease-modifying therapy. |
| --- |

## Slide 24
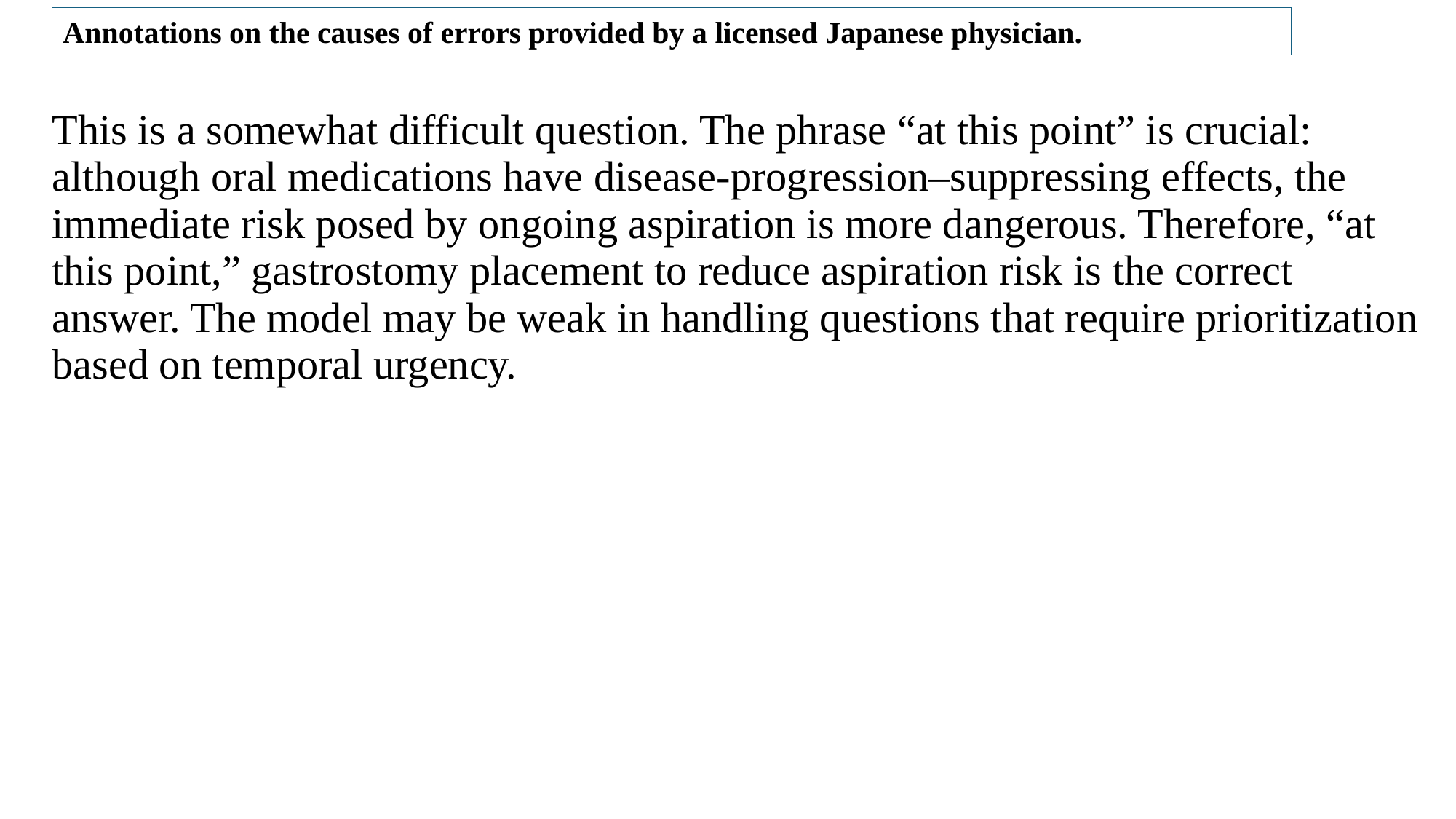

Annotations on the causes of errors provided by a licensed Japanese physician.
| This is a somewhat difficult question. The phrase “at this point” is crucial: although oral medications have disease-progression–suppressing effects, the immediate risk posed by ongoing aspiration is more dangerous. Therefore, “at this point,” gastrostomy placement to reduce aspiration risk is the correct answer. The model may be weak in handling questions that require prioritization based on temporal urgency. |
| --- |
